# Predicting New Anti-Norovirus Inhibitor With the Help of Machine Learning Algorithms and Molecular Dynamics Simulation–Based Model

**DOI:** 10.3389/fchem.2021.753427

**Published:** 2021-11-17

**Authors:** Oluwakemi Ebenezer, Nkululeko Damoyi, Michael Shapi

**Affiliations:** Department of Chemistry, Faculty of Natural Science, Mangosuthu University of Technology, Durban, South Africa

**Keywords:** anti-norovirus, hepatitis C virus (HCV), machine learning, molecular docking, molecular dynamics

## Abstract

Hepatitis C virus (HCV) inhibitors are essential in the treatment of human norovirus (HuNoV). This study aimed to map out HCV NS5B RNA-dependent RNA polymerase inhibitors that could potentially be responsible for the inhibitory activity of HuNoV RdRp. It is necessary to develop robust machine learning and *in silico* methods to predict HuNoV RdRp compounds. In this study, Naïve Bayesian and random forest models were built to categorize norovirus RdRp inhibitors from the non-inhibitors using their molecular descriptors and PubChem fingerprints. The best model observed had accuracy, specificity, and sensitivity values of 98.40%, 97.62%, and 97.62%, respectively. Meanwhile, an external test set was used to validate model performance before applicability to the screened HCV compounds database. As a result, 775 compounds were predicted as NoV RdRp inhibitors. The pharmacokinetics calculations were used to filter out the inhibitors that lack drug-likeness properties. Molecular docking and molecular dynamics simulation investigated the inhibitors’ binding modes and residues critical for the HuNoV RdRp receptor. The most active compound, CHEMBL167790, closely binds to the binding pocket of the RdRp enzyme and depicted stable binding with RMSD 0.8–3.2 Å, and the RMSF profile peak was between 1.0–4.0 Å, and the conformational fluctuations were at 450–460 residues. Moreover, the dynamic residue cross-correlation plot also showed the pairwise correlation between the binding residues 300–510 of the HuNoV RdRp receptor and CHEMBL167790. The principal component analysis depicted the enhanced movement of protein atoms. Moreover, additional residues such as Glu510 and Asn505 interacted with CHEMBL167790 via water bridge and established H-bond interactions after the simulation. http://zinc15.docking.org/substances/ZINC000013589565.

## Introduction

Noroviruses were known as “Norwalk-like viruses” in the past, and these viruses were ascertained in 1972 by Dolin et al. (1972) and Kapikian et al. (1972). Noroviruses are positive-sense single-stranded viruses classified into the family of *Caliciviridae* and genus *Norovirus*. The genome of human norovirus (HuNoV) is ∼7.7 kb dimension and systematized into three different open reading frames (ORF). ORF1 encodes a large polyprotein into nonstructural proteins. These include VPg-like protein, viral protease, and RNA-dependent RNA polymerase (RdRp). ORF2 encodes a major capsid protein 1 (VP1) that can self-assemble into virus-like particles (VLPs), and ORF3 encodes an insignificant but essential protein assumed to be involved in the building of progeny particles (Allen et al., 2008; Kim et al., 2015). Furthermore, HuNoV is the most common agent of viral gastroenteritis, causing about ∼700 million infections, 219,000 deaths, and $60 billion societal costs across the world annually in the recent decades (Bartsch et al., 2016; Van Dycke et al., 2021). Chronic and severe HuNoV infections are increasing as the immunocompromised population grows, for example, in transplant and cancer patients (Van Dycke et al., 2021), and HuNov also causes about one-fifth of all gastroenteritis cases in children less than five years worldwide (Lartey et al., 2020). Hence, there is a high demand for the efficient development of antiviral drugs or vaccines to prevent and treat norovirus disease. However, currently, no specifically approved medication or vaccine is available to fight against HuNoV infection. The RdRp is a crucial and attractive drug target for the development of anti-norovirus agents. It played an essential role in viral replication but has no host cell homologs; thus, effective HuNoV RdRp inhibitors can be developed with safer and more effective therapeutics for treating norovirus diseases. Based on the mode of action, RdRp inhibitors can be classified into nonnucleoside inhibitors (NNIs) and nucleoside or nucleotide inhibitors (NIs) (Wei et al., 2016; Ferla et al., 2018). NIs act as substrate imitators for the polymerase, blocking the replication and elongation of the RNA chain by competing with the natural nucleoside triphosphate (Ng et al., 2008; Wei et al., 2016). CMX521 (NI) was recently reported as the first direct-acting antiviral therapeutic for treating and preventing norovirus infections and advancing clinical trials (phase 1). The NIs that inhibit MNV and HuNoV replications but are unable to proceed to clinical trials include favipiravir (T-705) (Furuta et al., 2009), ribavirin (RBV) (Chang and George, 2007), 2′-C-methyl-cytidine (2CMC) (Rocha-Pereira et al., 2012; Kolawole et al., 2016), and 2′-fluoro-2′-C-methyl-cytidine (2′-F-2CMC) (Costantini et al., 2012), among others. Whereas the NNIs bind to the allosteric sites of RdRp, causing a change in the conformation necessary to initiate RNA synthesis and inhibit enzyme activity (Barreca et al., 2011; Wei et al., 2016; Bassetto et al., 2019). The NNIs include NAF2 (Tarantino et al., 2014), suramin, NF023 (Mastrangelo et al., 2012), PPNDS (Croci et al., 2014), NCI02, and NIC12 (Eltahla et al., 2014). Also, nitazoxanide (NNI), an agent with broad antimicrobial activity, has proven to be a therapeutic alternative for patients with norovirus gastroenteritis in clinical trials, but the specific mechanism of nitazoxanide is still unknown (Siddiq et al., 2011). In contrast, hepatitis C virus (HCV) is a small, enveloped virus of 50–80 nm diameter, also with a positive sense, single-stranded RNA (+ssRNA) like norovirus, and the RNA molecule contains a single open reading frame (ORF) but lacks a 5ʹ cap (Eltahla et al., 2015). Considering the similarities of the replication strategies between noroviruses and HCVs and the mode of action of NIs and NNIs in their RdRp binding pocket, the NNIs and NIs of HCV NS5B polymerase could serve as starting molecules or scaffolds for designing, synthesizing, and developing antiviral agents against norovirus infections. Although the agent that targets RdRp enzymes has been able to proceed to the clinical trial, sadly, no compound has been pinpointed as a specific inhibitor of the HuNoV RdRp either due to off-target, toxicity, or low human intestinal absorption of these compounds. Thus, there is still a great need for searching compounds with low toxicity and good bioavailability with minimal side effects that can be effectively developed into antiviral therapeutic agents targeting norovirus infection.

Furthermore, many computational chemists are exploring machine learning approaches due to their high accuracy in activity prediction across multiple targets and pharmacokinetic properties (Afanasyeva et al., 2020). Machine learning models could be beneficial for lead optimization and chemical compound prioritization when using computer-aided drug design (Lavecchia, 2015). Statistical learning algorithms, namely, Naïve Bayesian (Murakami and Mizuguchi, 2010; Fang et al., 2013) random forests (RFs) (Jayaraj et al., 2016; Wei et al., 2016; Li et al., 2019a; Wei et al., 2020), support vector machines (SVMs) (Han et al., 2008; Mahé and Vert, 2009; Fang et al., 2013; Jayaraj and Jain, 2019; Wei et al., 2019), decision stump (Nand et al., 2020), artificial neural networks (ANNs) (Lobanov, 2004; Li et al., 2019b), and k nearest neighbors (kNNs) (Mahé and Vert, 2009), have been used to build models and effectively employed in virtual screening, prediction of protein–protein interactions, ADMET prediction, and pharmacokinetic studies with substantial outputs. Kadioglu and co-workers applied a workflow of combined virtual drug screening, molecular docking, and supervised machine learning algorithms to identify novel drug candidates against COVID-19 (Kadioglu et al., 2021). Zhang et al. built a machine-learning–based scoring function for the effective virtual screening of lead compounds targeting the viral neuraminidase (NA) protein to develop novel anti-influenza therapies. The RF-NA-Score was detailed as the best model over the RF-Score, with a root-mean-square error of 1.46, Pearson’s correlation coefficient of 0.707, and Spearman’s rank correlation coefficient of 0.707 in a 5-fold cross-validation study (Zhang et al., 2017a). The best model was further used to virtually screen the SPECS database for NA inhibitors (Zhang et al., 2017a). In the reported work of Li et al., RF, SVM, kNN, and C4.5 decision tree models were used to discriminate inhibitors of the human topoisomerase I (Top1) protein from the non-inhibitors with total prediction accuracies ranging between 89.70 and 97.12% (Li et al., 2019a). Among machine learning algorithms, the RF model was detailed as the best model and was used to virtually screen the Maybridge database for Top1 inhibitors. But, until now, there is limited investigation on classification predictions of HuNoV RdRp inhibitors and noninhibitors.

In this study, we conducted a machine learning model combined with molecular docking and molecular dynamics simulation to identify small molecule inhibitors of HCV that could potentially target HuNoV RdRp and could be further developed into an anti-norovirus agent. This is the first report that used automated learning approaches, validated, and demonstrated a virtual screening model to identify HuNoV RdRp inhibitors to the best of our knowledge. Naïve Bayesian and random forest models were built to categorize norovirus RdRp inhibitors from the non-inhibitors using their molecular descriptors and PubChem Fingerprints. The best model observed has accuracy, specificity, and sensitivity values of 98.40%, 97.62%, and 97.62%, respectively. Meanwhile, an external test set was used to validate model performance before the model’s applicability to the screened HCV compounds database. As a result, 775 compounds were predicted as NoV RdRp inhibitors. The predicted active compounds with drug-likeness properties were docked into the binding site of the HuNoV RdRp protein. The protein–ligand complexes were further subjected to MD simulations to investigate the dynamic nature of the ligand with the protein during the 50 nanosecond (ns) simulation process.

## Materials and Methods

### Data Source and Dataset Pre-Processing

A total of 188 compounds of norovirus RdRp were collected and downloaded from the BindingDB (Liu et al., 2007) and PubChem databases (bioassay, 2013) for the training set in the machine learning model. The data sets were divided into an active set (65 compounds with inhibitory activity of <50 µM) and an inactive set (123 compounds with inhibitory activity of ≥50 µM). Besides, a separate dataset containing 40 compounds was used as an external dataset. Molecular descriptor parameters were divided into 70/30% for a training and testing data set to evaluate model performance. Furthermore, virtual screening was performed on HCV NS5B RNA-dependent RNA polymerase (HCV NS5B RdRp) compounds containing diverse scaffolds and substituents from the ChEMBL database (1766 compounds). In addition, before calculating the molecular descriptors and fingerprints, duplicates, missing canonical smiles, and bioactivity values were removed; all the inorganic counterions were filtered out for easier handling in PaDEL-Descriptor software (Yap, 2011).

Furthermore, all compounds were advanced to chemical descriptor computation. The 1D, 2D, and fingerprint (PubChem) were generated as an input for the machine learning model in Weka software (Witten and Frank, 2002). A total of 1,444 descriptors were calculated using WEKA by employing 489 atom type electro-topological state indices(Hall and Kier, 1995), 96 burden modified eigenvalues (Todeschini and Consonni, 2009), and 346 2D autocorrelation (Todeschini and Consonni, 2009). In addition, 43 extended topochemical atoms (Roy and Ghosh, 2004), 21 topological charge (Todeschini and Consonni, 2009), 68 ring counts, molecular linear free energy relation (Platts et al., 1999), average molecular weight, 91 descriptors based on Barysz matrix, 32 chi path descriptors, 12 constitutional descriptors were also used to calculate decriptors. Besides, PubChem fingerprint encrypted molecular fragment information with 881 binary digits (Cereto-Massagué et al., 2015).

### Prediction Method for Model Building Using Machine Learning Approaches

#### Naïve Bayes

The Naïve Bayes algorithm is an unsophisticated likelihood, rapid, precise, reliable and robust classifier (Kohavi, 1996). The approach used the Bayes theorem to adopt classification with unbiased attributes. Furthermore, the algorithm is a simple probabilistic classifier and presupposes the independence of features of a given class which can significantly diminish the complexity of the development of the classifier (Murakami and Mizuguchi, 2010). Besides, it can categorize active compounds from inactive compounds and minimize the odds of misclassification. The model used classification algorithms for data distribution according to multivariate Bernoulli distributions, which means there were numerous features in a data set. However, each one is presumed to be a binary-valued (Bernoulli, Boolean) variable. The sample features are caused to employ the model, and the weight is computed for each feature using a Laplacian-adjusted probability estimate. The weights are summed to present probability approximate, which is a relative predictor of the likelihood of that sample in the active subset (Fang et al., 2013).

#### Random Forest

The random forest (RF) classifier is an ensemble modeling method that combines many tree-like predictors as base learners. In this approach, the bagging inkling is used in sequence with random feature selection (Zhang et al., 2017b). A different training set is drawn, with replacement, from the original training set. Then, a tree is grown on the new training set using random feature selection, whereas the trees grown are not pruned (Cutler et al., 2012). The bagging idea is employed to boost precision when random features are used. Besides, it can give continuing estimates for the generalization error (PE∗) of the combined ensemble of trees and the estimates for the strength and correlation (Cutler et al., 2012). The correctness of random forest is as good as AdaBoost and occasionally improves and gives valid internal estimates of error, strength, association, and variable importance. Besides, it is straightforward, easily parallelized, and uses more variables than samples. Even if the data is fused with noise or not sensitive to algorithmic parameters, it has an excellent predictive ability (Zhang et al., 2019). Since RF integrates many simple models, it can effectively reduce over-fitting problems. RF can also handle both categorical and continuous variables, which can return the importance of variables and be freely implemented with a high quality.

#### Validation of the Model Performance of the Naïve Bayesian and Random Forest Approaches

The equation below presented the parameters used to quantify the superiority of the NB and the RF classifiers. These parameters are true positives (TP), true negatives (TN), false positives (FP), false negatives (FN), sensitivity (SE), specificity (SP), the overall prediction accuracy (Q), and Matthew’s correlation coefficient (MCC). TP denotes the number of active compounds that are predicted as norovirus inhibitors. TN denotes the number of inactive compounds that are predicted as inactive compounds. FP stands for the number of inactive compounds predicted as norovirus agents, and FN is the number of norovirus agents predicted as inactive compounds. SE denotes the prediction accuracy for norovirus agents, which means the number of true positive tests. SP denotes the prediction accuracy for the non-norovirus agents, which means the number of true negatives that test negative, and specific tests will not yield false positives or misclassify.

$$SE=\;\frac{TP}{TP+FN};$$ (1)

$$SP=\;\frac{TN}{TN+FP\;};$$ (2)

$$Q=\frac{TP+TN}{TP+TN+FP+FN};$$ (3)

$$MCC=\;\frac{TP\;\times TN\;-FN\;\times FP}{\sqrt{\left(TP+FN\;\right)\left(TP+FP\right)\left(TN+FN\right)\left(TN+\;FP\right)}\;\;\;\;\;\;\;\;\;\;\;\;\;}.$$ (4)

### Lipinski’s Rule of Five and ADMET Properties

Data warrior software is accustomed for data exploration and visualization. The built-in cheminformatics algorithms in the software make it a flexible tool for exploring large data sets of chemical structures with alpha-numeric properties (Sander et al., 2015). It can be used to determine physicochemical properties, lead- or drug-likeness–related parameters, ligand efficiencies, various atom and ring counts, molecular shape, flexibility and complexity, and indications for potential toxicity in a compound. The Lipinski’s rule of five (Lipinski et al., 1997) was predicted using this software. Five significant parameters were estimated directly from the chemical structure. The total molecular weight in g/mol, CLogP [conc(octanol)/conc(water)], CLogS (water solubility in mol/L), H-bond acceptors (HBA), H-bond donors (HBD), and topological polar surface area (TPSA) using the Ertl approach (Ertl et al., 2000). The output compounds from Lipinski’s rule of five were subjected to ADMET (absorption, discretion, metabolism, and toxicity) prediction analysis using the pkCSM (Pires et al., 2015) web server tool. A compound’s pharmacokinetics is crucial in developing drugs because numerous compounds analyzed in clinical trials lost their way out to the market due to insufficient efficacy or obnoxious side effects.

### Molecular Modeling

#### Ligand and Protein Preparation

The compounds that passed through Lipinski’s rule of five were run using OMEGA python application in OpenEye Software to generate 300 conformers for each molecule. The OMEGA tool stipulates torsion driving and distance geometry for the conformational cohort. The torsion driving method works best on molecules that have a small, flexible ring. In contrast, the distance geometry method works for all molecules and is also designed for large, flexible rings (macrocycles) (Hawkins et al., 2010). Meanwhile, generating the conformers before the molecular docking leads to decrease in time for the protein–ligand docking. Spruce (Spruce 1.3.0.1: OpenEye S, 2021) was used to prepare the HuNoV RdRp protein downloaded from the protein data bank (PDB). The protein preparation workflow is as follows: 1) expansion of the asymmetric unit to the biological of the x-ray crystallography; 2) enumeration of alternate locations; 3) building missing side chains, capping chain breaks, and modeling of the missing loops; 4) placement and optimization of hydrogen atoms including tautomer enumeration of ligands and cofactors, and evaluation of those tautomer states in the biomolecule structure. Furthermore, there were no constraints specified; thus, the prepared protein was saved for further analysis.

### Molecular Docking

In the FRED’s docking procedure (OEDOCKING, 2014), the HuNoV RdRp protein structure and the multi-conformer ligands generated from the OMEGA application were used as the inputs for docking. The first step was the exhaustive search, whereby each ligand conformation is analytically rotated and translated within the protein’s binding site at a resolution of 1 Å. The false poses were dropped during the search, and the remaining poses were scored. Then, we continued with the subjection of the scored poses to optimization, and every pose that passed a bump check was scored. The top-scoring poses were improved by observing nearby rotations and translations at a resolution of 0.5 Å across all ligand conformers. Chemgauss4 that accounts for hydrogen bond interactions, metal-chelator interactions, de-solvation effects, and the shape complementarity of the ligand to the active site was employed as the scoring function (McGann, 2012).

#### Molecular Dynamics Simulations

The MD simulations were achieved using the Desmond simulation package (Bowers et al., 2006) to explain these compounds’ superiority against HuNoV RdRp. The system was built using a pre-defined TIP3P water model. This was structured under periodic boundary conditions at distances of 10 Å units; meanwhile, the ligands and the proteins were first prepared using the OPLS-2005 force field. The complexes’ charge was neutralized with balancing Na^+^/Cl^−^ ions, and the system minimized their energies by heating and equilibrium processes before the MD simulations. The NPT ensembled with the temperature of 300 K, and a pressure 1 bar was utilized in all the runs. The simulation length was 50 ns with a relaxation time of 1ps for the ligands. The interactions of the protein–ligand complexes were analyzed using the simulation interaction diagram tool in the Desmond package (DESR, 2021). The protein–ligand complexes’ dynamical properties were detailed by observing the root mean square deviation (RMSD) and root mean square fluctuation (RMSF).

## Result and Discussion

### Analysis of the Chemical Space

The physicochemical properties of the training and external datasets were calculated using DataWarrior software (Sander et al., 2015). The software is used for chemical data exploration and visualization. We used the calculated values of MW and CLogP to observe the diversity of the chemical space of the training set (inhibitors and non-inhibitors of NoV RdRp) and virtual screening (inhibitors and non-inhibitors of HCV). There is always a drawback in the machine learning model approach when the dataset of the compounds is not adequately dispersed. The graphical representation of the chemical space distribution showed that the total molecular weight (MW) was between 149.00–980.00 and CLogP was from 5.00to6.30 for the training dataset (Supplementary Figure S1A). Also, the chemical space distribution for the external datasets ranged from 180.00–1800 and 4.00–17.00 for MW and CLogP, respectively (Supplementary Figure S1B). The distribution of MW and CLogP values in both datasets indicated that the compounds occupied substantial chemical space. For the machine learning model construction, the compounds targeting NS5B RdRp of HCV were selected as the training set.

### Evaluation of Model Prediction

Two types of descriptors were used for modeling in this study, 1D/2D and PubChem fingerprint. Various descriptors, including acidic group count, ALOGP, APol, ALOGP, bond count, carbon types, HBA, HBD, Lipinski’s rule of five, rotatable bonds count, autocorrelation, TPSA, van der Waals volume, were used. A total of 1,444 descriptors were produced, including 881 PubChem fingerprints (PFPs) were calculated using PaDEL software. The training set was filtered by removing descriptors with missing parameters. This step automatically removed all continuous attributes and the descriptors that were not valuable for the classification. Furthermore, two classification models were explored by using NB and RF classifiers ***.*** The models were initially performed on the fingerprint, as shown in Table 1. The fingerprint presents the chemical information in any chemical structures in binary vectors. The result from the fingerprint detailed that the performance of the RF classifier was superior to the performance of the NB classifier. For example, the ROC of NB is 0.879, while RF is 0.997. In addition, the SE (95.38%), the SP (97.56%), and the overall accuracy (96.80%) of RF are superior to the statistical values from NB (SE = 81.43%, SP = 81.25%, and Q = 81.83%). The correctly classified instances (TP + TN) of the NB classifier were 153 out of 188 sum weights.

**TABLE 1:** Performance of the Naïve Bayes and random forest classifiers.

**Training set**														
**Descriptors**	**Models**	**CI**	**IN**	**TP**	**FN**	**FP**	**TN**	**KS**	**RMSE**	**MAE**	**ROC**	**SP (%)**	**SE (%)**	**Q (%)**
PFP	NB	153	35	39	9	26	114	0.561	0.932	0.186	0.879	81.43	81.25	81.38
	RF	182	6	62	3	3	120	0.930	0.174	0.099	0.997	97.56	95.38	96.80
PFP_1 and 1D/2D	NB	159	29	53	17	12	106	0.667	0.393	0.154	0.857	89.83	75.71	84.57
	RF	185	3	62	0	3	123	0.964	0.141	0.085	0.999	97.62	100.00	98.40
**Percentage splitting (70/30)**														
**Descriptors**	**Models**	**CI**	**IN**	**TP**	**FN**	**FP**	**TN**	**KS**	**RMSE**	**MAE**	**ROC**	**SP (%)**	**SE (%)**	**Q (%)**
PFP	NB	43	13	6	5	8	37	0.333	0.481	0.250	0.758	91.43	37.50	74.51
	RF	42	13	11	10	3	32	0.469	0.504	0.261	0.875	91.43	52.38	76.79
PFP_1 and 1D/2D	NB	42	14	9	9	5	33	0.391	0.500	0.231	0.773	86.84	50.00	75.00
	RF	43	13	9	8	5	34	0.422	0.508	0.268	0.881	87.18	52.94	76.79

TI—Total number of instances; CI—Correctly classified instances; IN—Incorrectly classified instances; TP—True positive; FN—False negative; FP—False positive; KS—Kappa statistics; RMSE—Root mean squared error; MAE—Mean absolute error; SP—Specificity (%); SE—Sensitivity (%); Q—Accuracy (%).

In contrast, the RF classifier generates a total of 182 correctly classified instances. Also, the kappa statistic (KS) for the RF model is 0.930 and the RMSE value is 0.174, while the NB model generates a kappa statistic value of 0.561 and RMSE value of 0.932. The kappa statistic measures the reliability between the actual values of the instance to be classified and the classifier model. Thus, the kappa statistic for the RF classifier is suggested to be in almost perfect agreement, this is otherwise for the NB classifier, which is suggested to be in moderate agreement.

The combination of fingerprint and 1D/2D descriptors significantly improved the model performance accuracy, as shown in Table 1. Using RF and NB classifiers with the same descriptors, the performance of the RF classifier is superior to NB. The accuracy of NB increased from 81.83 to 84.97%, and the ROC value decreased from 0.879 to 0.857. There was a significant decrease in the RMSE (0.154) of the NB classifier, and the kappa statistic increased to 0.667. Notably, if the 1D/2D descriptors were removed, none of the classifiers had superior predictive power. There was also an increase in the accuracy of the RF classifier (98.40%), whereas the SP (97.62%) and SE (100%) calculated were superior to the NB classifier. The RF classifier was identified as the best model, with better accuracy (98.40%), RMSE (0.141), and MAE (0.085) values. Molecular descriptor parameters were divided into 70/30% for the training and testing data sets to evaluate model performance. As shown in Table 1, the best model for predicting inhibitors of NoV RdRp, that is, the RF classifier displayed the highest SE, SP, and Q values (52.94%, 87.18%, and 76.79%, respectively) for 1D/2D descriptors. To further prove the performance of the models, an external dataset (27 active compounds and 13 inactive compounds) of norovirus RdRp was investigated. The RF classifier was identified as the best model with an accuracy of 100% for both descriptors (Supplementary Table S1). Thus, the RF classifier was further used to identify potential NoV RdRp by exploring the NS5B RdRp HCV dataset (1,289 compounds) in the CHEMBL database. The RF model predicted 775 compounds as potential inhibitors of NoV RdRp. The statistical performance values for both models are listed in Supplementary Table S1. The KS value for the RF model was 0.997, and the RMSE value was 0.110. In addition, the ROC value was 1.00, which signified the perfect distribution of the active compounds from inactive compounds without overlapping, whereas 99.80%, 99.87%, and 99.84% were the values detailed for SE, SP, and Q, respectively.

### Pharmacokinetic Properties

Moreover, 775 compounds were filtered using Lipinski’s rule of five since the method could rapidly filter out probable challenging molecules. The compounds that fell out of the five rules were quickly dropped, and the remaining compounds (352) were used for further analysis. Surprisingly, out of the 352 well-thought-out compounds for the ADMET properties, only 59 compounds were of good pharmacokinetic after considering properties such as water solubility, caco_2_ permeability, intestinal absorption (human), skin permeability, blood−brain barrier (BBB) permeability, total clearance, AMES toxicity, hERG I inhibitor, and hERG II inhibitor (Supplementary Table S1). Predicting ADMET properties is essential in drug development because it helps in the significant removal of the compounds that will not meet the optimal requirement of the druggable agent.

### Similarities Between HuNoV and HCV Sequences/Description of HuNoV RdRp

**FIGURE 1:**
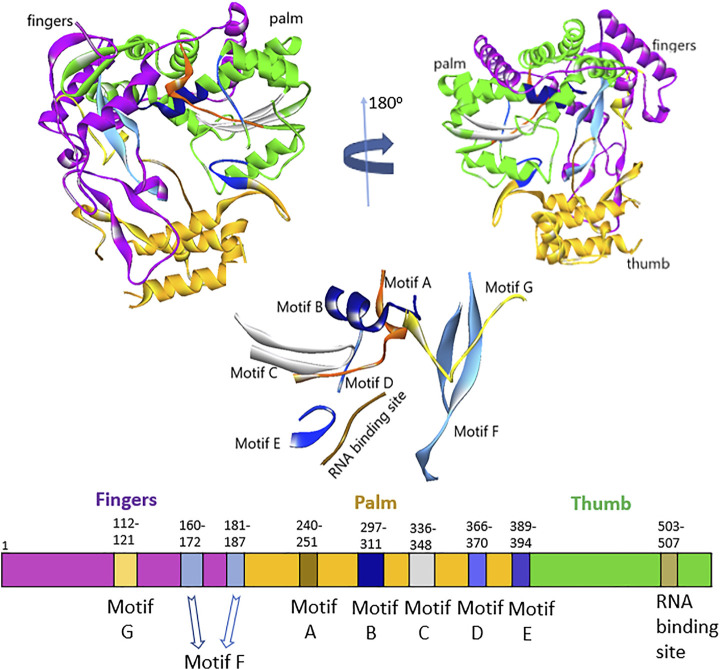
Representation of fingers, palm, and thumb domains, including seven motifs **(A–G)** HuNoV RdRp.

Sequence analysis of HuNoV (RCSB, 2014) and HCV (Di Marco et al., 2005) showed about 16% sequence identity, but interestingly, they still had specific architecture enzymatic mechanisms. The residues lining the binding pocket of HuNov RdRp that were conserved across the HCV were T419, R414, S503, G509, and V510 (Supplementary Figure S2). The five steps that occurred in RNA synthesis by the norovirus RdRp active site were as follows (Choi, 2012): 1) VPg uridylation by the 3D polymerase (initiation) and 2) nucleotidyl reaction, which involves different steps: 1) polymerase first binded a template–primer; 2) binding of an NTP complementary to the template base; 3) conversion of polymerase complex into an activated form, that includes a conformational change from an “open” to a “closed” form; 4) nucleotidyl transfer reaction; 5) release of pyrophosphate product 6) template–primer translocation. The fingers, palm, and thumb were the three main domains of HuNoV RdRp, including seven motifs (A–G) (Figure 1). The N-terminal domain bridged the fingers and thumb domains. The fingers domain consists of two motifs (F and G), whereas the palm domain, the catalytic core of polymerase, embraces four highly conserved sequence motifs (A–D) (Deval et al., 2017; Smertina et al., 2019). The fingers domain plays a crucial role in setting the geometry of the active site (Figure 1). At the same time, the thumb domain consists of residues that involve packing against the template RNA and stabilizing the initiating NTPs on the template (Ng et al., 2008).

The thumb domain consists of motif E and the enzyme active site. The active site of RdRp located at the thumb domain consists of three conserved Asp residues essential for mediating catalysis through a two-metal-ion mechanism. The amino acids, Arg, Asn, and Ser, are other vital residues required for substrate binding and catalysis (Zamyatkin et al., 2008; Prasad et al., 2016; Venkataram Prasad et al., 2016). The palm and thumb domains constitute site B of the protein structure. The two Asp residues position in the motif C interact with two divalent metal ions to achieve the nucleophilic attack, allowing the incoming ribonucleotide to the RNA chain. Also, in the motif D, the lysine residue acts as the general acid that deprotonates the pyrophosphate leaving group and influences the amount of nucleotide addition. The only glycine in the motif D of the palm domain helps as a hinge for the structure that might play a critical role in its conformational changes (Jácome et al., 2015). The motif F, which is highly conserved, comprises the positively charged residues Arg and Lys that facilitate interaction with α- and β-phosphates of the incoming NTP, likely to stabilize the pyrophosphate as a leaving group (Deval et al., 2017). Motifs B and G coordinate template and primer binding and motifs A and C execute the catalysis of nucleotide binding. The motif G is located in the template cleft and is involved in protein primer orientation during the initiation of RNA replication (Ng et al., 2008; Smertina et al., 2019). Also, the motif H lacks the presence of conserved amino acids; the motif is established based on multiple sequence alignments, but its actual function has not been reported (Černý et al., 2014).

### Protein Template Search

Different crystal structures of HuNoV RdRp have been deposited in the protein data bank (PDB). The sequence of HuNoV RdRp (Entry—D0UGI3; Protein—NTPase; Gene—RdRp) was downloaded from the UniProt database (Consortium, 2012) to search for a suitable protein for molecular docking. The sequence was further imported into the maestro interface, and the BLAST option in the homology modeling of the Prime application (Zhu et al., 2014) was used to blast the sequence. Comparing the sensitivity search and the alignment accuracy using different protein similarity scoring matrices such as BLOcks SUbstitution Matrix (BLOSUM45, 62, and 80) and point accepted mutation matrix (PAM40 and 70) were considered. Interestingly, all the substitution matrices generated similar results except PAM40, which showed that the templates 1SHO, 1SH2 and, 1SH3 were ranked top before 3H5X, 2B43, 4LQ3, 4LQ9, and 4NRT (Table 2). An insignificant difference was observed comparing the similarity and identity value of all the templates.

**TABLE 2:** Details of different substitution matrices result in finding a template for the HuNoV RdRp sequence.

Substitution matrix	Templates
PAM40	3H5X, 1SH0, 1SH2, 1SH3, 2B43, 4LQ3, 4LQ9, 4NRT
PAM70	1SH0, 1SH2, 1SH3, 3H5X, 2B43, 4LQ3, 4LQ9, 4NRT
Blosum45	3H5X, 1SH0, 1SH2, 1SH3, 2B43, 4LQ3, 4LQ9, 4NRT
Blosum62	3H5X, 1SH0, 1SH2, 1SH3, 2B43, 4LQ3, 4LQ9, 4NRT
Blosum80	3H5X, 1SH0, 1SH2, 1SH3, 2B43, 4LQ3, 4LQ9, 4NRT

The crystal structure of 4LQ3 with its co-crystallized ligand, namely, pyridoxal-5′ -phosphate-6-(2′ -naphthylazo-6′ -nitro-4′,8′ -disulfonate) tetrasodium salt (PPNDS), was downloaded from the protein data bank (RCSB, 2014). PPNDS was first known as the P2 receptor antagonist and represented the pyridoxal-5′-phosphate analog with the superior activity at P2X_1_ receptors (Lambrecht et al., 2000). It was reported that PPNDS was a potent inhibitor of human and murine norovirus RdRp. Furthermore, PPNDS, the co-crystal ligand of 4LQ3, emerged as the most potent compound in the inhibitory activity of HuNov RdRp with an IC_50_ value of 0.45 μM. In contrast, NAF2, the co-crystal ligand of 4LQ9, inhibited HuNov RdRp activity with an IC_50_ value of 14 μM. In addition, PPNDS is capable of binding to the free enzyme together with the enzyme–substrate complex. The binding site of PPNDS (site-B) is structurally equivalent to the binding site of benzothiadiazine inhibitors (palm I site) in the HCV RdRp (Tarantino et al., 2014). The site-B is within the thumb domain, closer to the C-terminal of the HuNoV RdRp, reports for taking part in the initiation of RNA replication (Tarantino et al., 2014; Zhang et al., 2019). PPNDS assists in fixing the C-terminal end of the enzyme within the active site, possibly preventing the access of both the ssRNA template and the NTPs. (Croci et al., 2014; Tarantino et al., 2014). Unfortunately, negative charges on the sulfonate group on PPNDS have led to its poor cell absorption. Meanwhile, a new drug’s absorption properties are crucial, enabling the drug to penetrate the cell to reach the target site in reasonable concentrations, and create the physiological effect with minimal or no side effects. Hence, PPNDS could not proceed to clinical trials due to the deficiency in its drug-likeness properties. The binding pocket of PPNDS in HuNoV RdRp was the target for anti-norovirus agents that have good pharmacokinetic properties and with high chances of blocking the RNA viruses require further exploration.

### Molecular Docking

**FIGURE 2:**
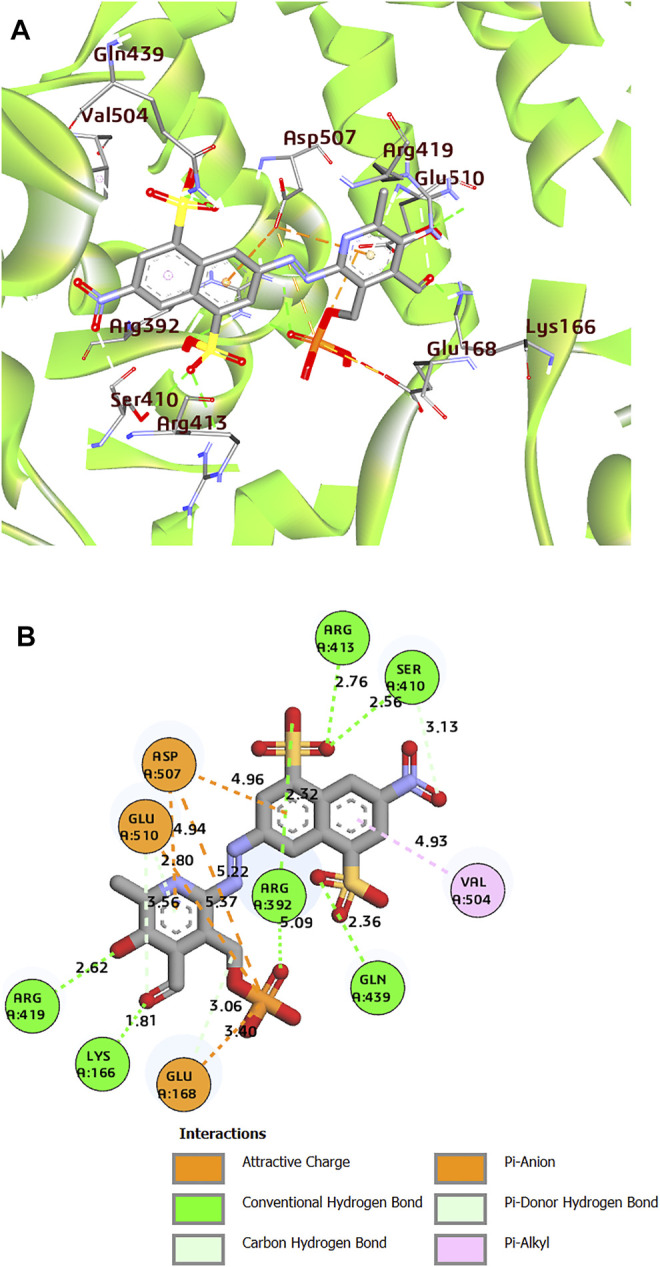
**(A)** 3D representation of docked complex of PPNDS-HuNoV RdRp (The multicolor sticks represent the interacting residues in the binding site of HuNoV RdRp. The big stick color represents the PPNDS molecule). **(B)** 2D representation of the protein–ligand complex of PPNDS including distances between the ligands and the interacting residues.

**FIGURE 3:**
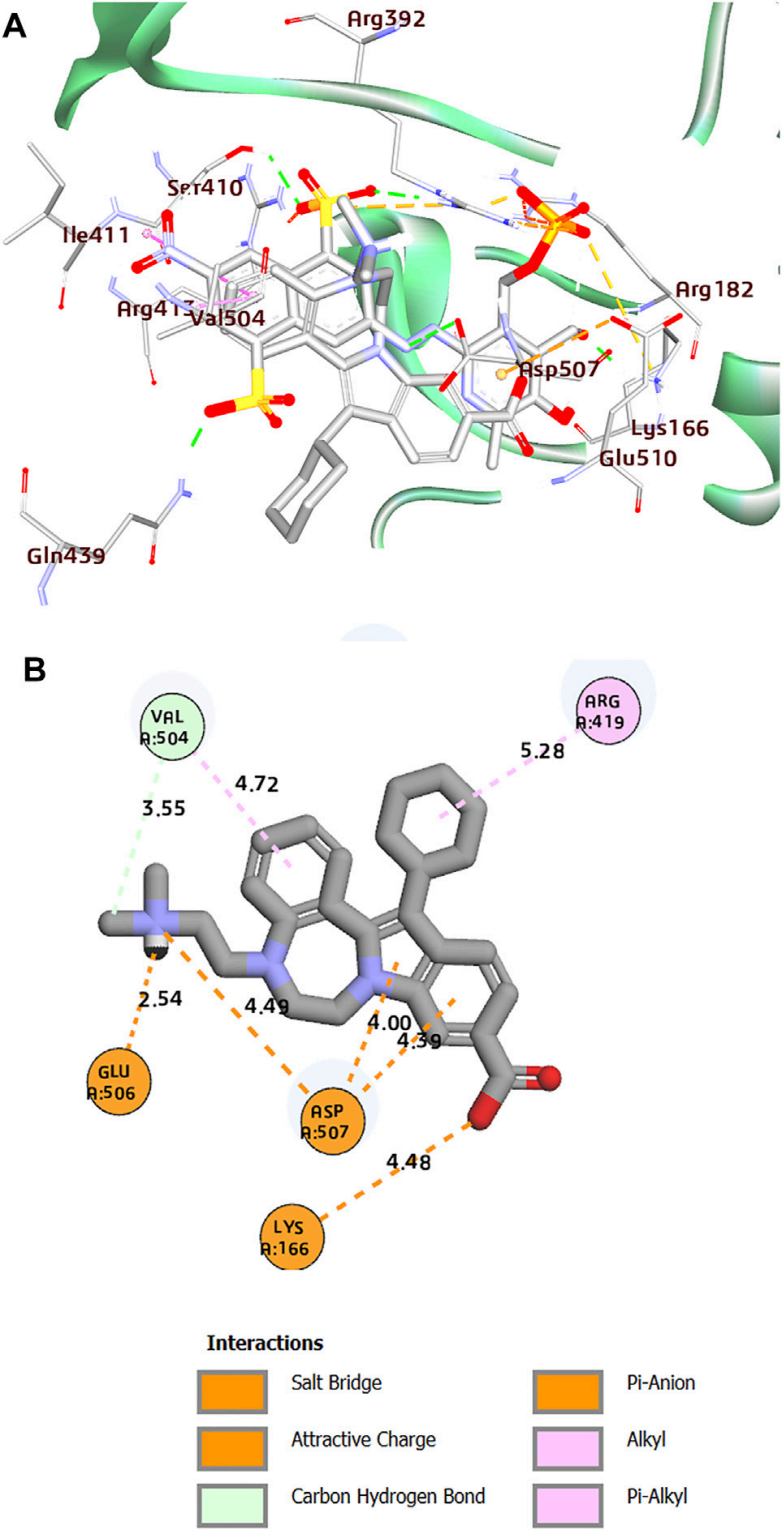
**(A)** Superposition of co-crystal structures of PPNDS and CHEMBL1204385 along with their interactions in the binding site of HuNoV RdRp (PDB: 4LQ3). **(B)** 2D representation of the protein–ligand complex of CHEMBL1204385 including distances between the ligands and the interacting residues.

**FIGURE 4:**
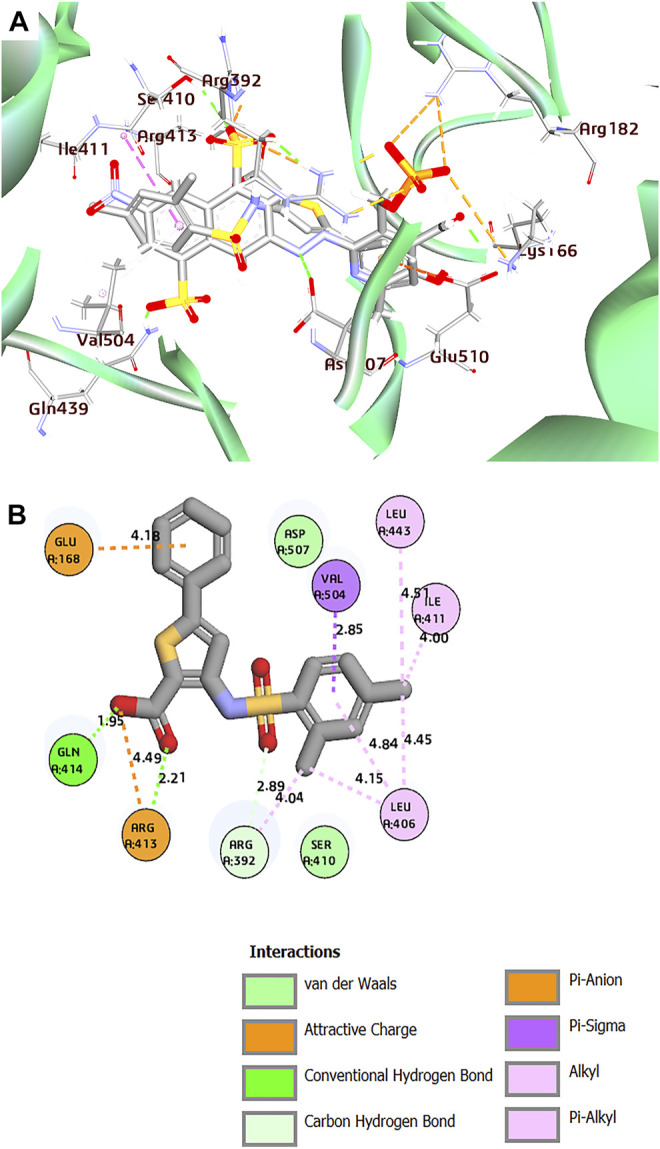
**(A)** Superposition of co-crystal structures of PPNDS and CHEMBL167790 along with their interactions in the binding site of HuNoV RdRp (PDB: 4LQ3). **(B)** 2D representation of the protein–ligand complex of CHEMBL167790 including distances between the ligands and the interacting residues.

Moreover, the three-dimensional protein structure of HuNoV RdRp downloaded from the PDB was prepared using the Spruce application (Spruce 1.3.0.1: OpenEye S, 2021). The co-crystal ligand, PPNDS, was further re-docked into the receptor to validate the receptor’s binding site using FRED. As shown in Figure 2, the native ligand formed interesting interactions with different residues in the receptor’s palm and thumb domains, including hydrogen bonds with residues Arg419, Arg392, Ser410, Glu510, Lys166, Gln439, and Arg413. The RNA binding site residue Asp507 was inserted between the naphthalene ring and the pyridine moiety of PPNDS to form an electrostatic interaction, while Val504 hydrophobically interacted with the pyridine ring. The predicted FRED Chemgauss4 score was 10.50 kcal/mol. Two compounds were found to have an average Chemgauss4 score but possessed better human absorption than the native ligand. The docked complex of the two compounds showing the best Chemgauss4 score are shown in Figures 3, 4, and the type of interactions with active residues are listed in Table 2.

**FIGURE 5:**
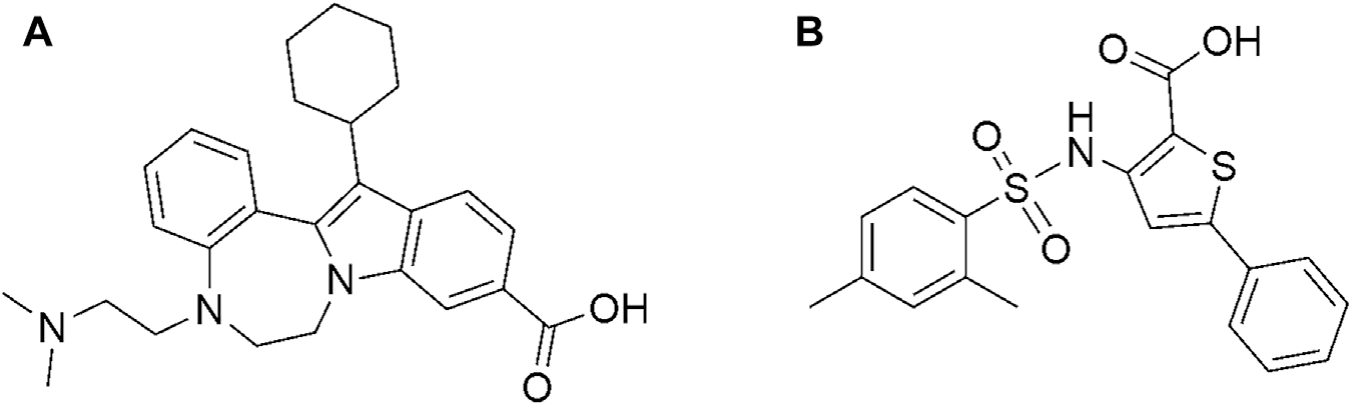
Chemical structure of **(A)** CHEMBL1204385 and **(B)** CHEMBL167790.

Interestingly, the two compounds have a similar network interaction landscape with PPNDS. However, the native ligand formed more hydrogen bonds with the active residues than CHEMBL1204385 and CHEMBL167790, as shown in Table 3. The visual detail of docked complexes of the two compounds showed no interaction with the metal ion. In the docked complex of CHEMBL1204385, the molecule was well positioned to allow interaction with the side chain of Glu506 and interaction with the compound’s secondary amine. As a result, the conformation adopted in this complex appeared to form better π-anion with Asp507 and π-alkyl with Val504 (Figures 3A,B). The oxygen atom of the carboxyl function group on the indole ring formed a hydrogen bond with Glu510. Few variations in the conformation and position of CHEMBL1204385 at the active site of HuNoV RdRp were detected compared to CHEMBL167790. CHEMBL167790 was more fitted into the binding site of HuNoV RdRp (Figure 4A). The capped end of Arg413, which is the guanidinium group, was protonated (positively charged). This led to attractive charge interaction with the carboxyl functional groups (negatively charged oxygen atom) of CHEMBL167790. The hydrogen bonding interaction was observed between amino acid Arg413 and the oxygen atom double-bonded to the carbon atom of the carboxyl functional group on the thiophene ring of CHEMBL167790. 3,4 -dimethyl phenyl well fitted into the hydrophobic pocket of the protein and formed interaction Leu406, Ile411, Leu443, and Arg392 (Figures 4A,B). From the visualization of the two complexes, it is well noted that hydrophobic, hydrogen bonding, and electrostatic interactions played a vital role in enhancing the thermostability of the protein–ligand complexes. Despite the binding of the ligands in the same binding landscape as the co-crystal ligand in the active pocket of HuNoV RdRp, interactions of few residues were not observed. This was due to the different orientation of the docked ligands, shape, and size compatibility compared to those of the co-crystal ligand. The chemical structures of the best two compounds from the molecular docking analysis are shown in Figure 5.

**TABLE 3:** Chemgauss4 score and interaction types of the two best compounds from the FRED docking.

ChEMBL ID	Chemgauss4 Score	Hydrophobic		Hydrogen bonds		Electrostatic			
		Pi-alkyl	Alkyl	Conventional H-Bond	Carbon H-Bond	Attractive charge	Salt bridge	Pi-anion	Pi-sigma
CHEMBL1204385	10.00	Val504	Arg419		Glu510, Asp507, Val504	Glu506		Asp507	
						Asp507, Lys166			
CHEMBL167790	10.39	Leu406	Leu406, Ile411, Leu443, Arg392	Arg413, Gln414	Arg392, Arg413	Arg413		Glu168	Val504

### Molecular Dynamics Simulations

**FIGURE 6:**
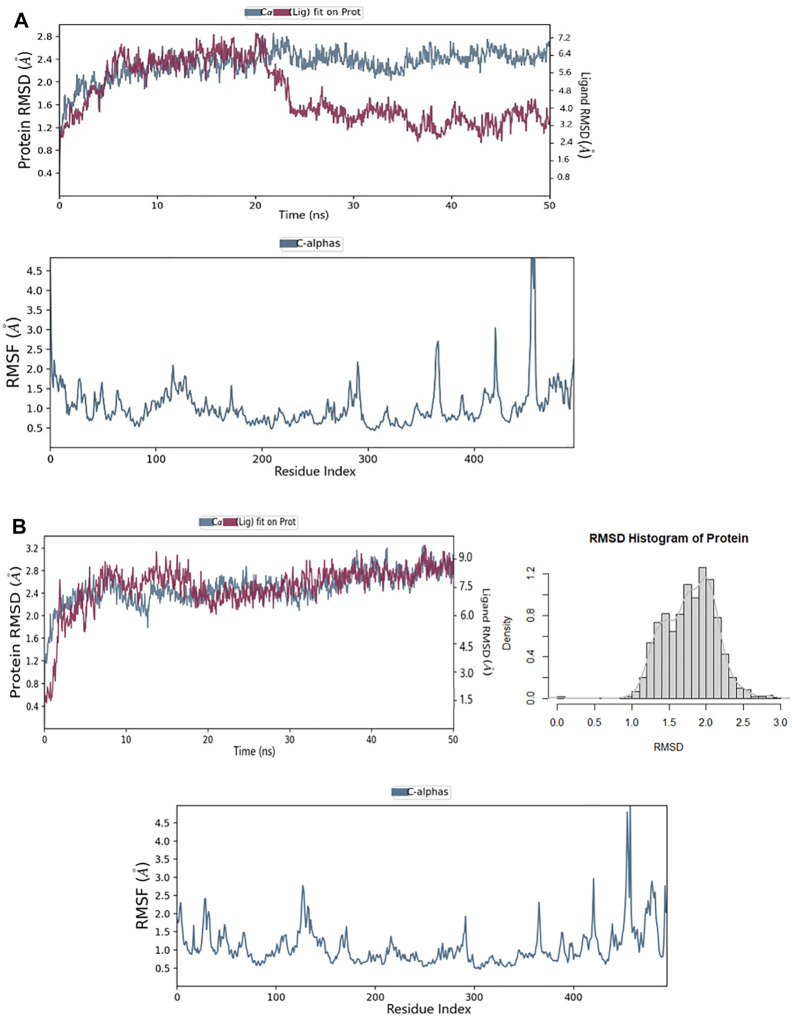
**(A)** RMSD and RMSF of the PPNDS complex after molecular dynamics **(B)** RMSD and RMSF of CHEMBL167790 complex after molecular dynamics.

The RMSD for the ligands with the target HuNoV RdRp receptor were depicted to show the thermal stability of the ligands to the targeted protein. Initially, the PPNDS–protein complex showed no variations at the first 20ns run simulation while high fluctuations were observed after 20 ns, and this continued till the end of the simulation. The protein plateaus at 1.2 Å were with an average RMSD of 2.4 Å and a maximum of 2.8 Å; for ligand, the RMSD was between 3.2 and 7.2 Å, as shown in Figure 6A. The protein in the CHEMBL167790 complex plateaus at 0.4 Å was with an average RMSD of 1.7 Å and a maximum of 2.90 Å; for ligand, the RMSD was between 0.8 and 9.0 Å. As established by the density versus RMSD histogram plots, conformational changes of the protein during the simulation period were minimal (Figure 6B). The RMSD of the ligand fluctuates at the start of the simulation, and there was only a slight fluctuation between 10 and 18ns. In addition, the change in the ligand RMSD is similar to the RMSD of the protein during simulation time, thus indicting the ligand stability in the protein active site. Besides, the RMSF of the ligands is needed further to evaluate their stability in the protein’s active and pinpoint possible binding modes. If the ligand is not stable, there will be large fluctuations in the RMSF; thus, we plotted the RMSF graph of the ligand, and it indicated the stability of the CHEMBL167790 with a single binding mode during the 50ns simulation time. The RMSF result of CHEMBL1204385 (Figure 6A) reflects high fluctuations at the loop region of the protein. In particular, the overall MD results show that the fluctuations of CHEMBL167790 (Figure 6B) are considerably better than PPNDS, and this shows the significance of CHEMBL167790 above PPNDS (Supplementary Figure S3).

After the molecular dynamic simulations, interactions of Leu443, Arg392, Gln414, and Val504 with CHEMBL167790 in the active pocket of HuNoV RdRp were preserved. Meanwhile, Leu406, Ile411, Arg413, and Glu168 were lost after the MD calculation (Supplementary Figures S4A, B). Additional residues such as Glu510, Asp419, and Asn505 interacted with the compound CHEMBL167790 and established H-bonding after the simulations.

Notably, CHEMBL167790 binded to the thumb domain and interacted with the residues Gln414 and Asp419; this result corroborated with the previous studies that showed JTK-109 (HCV NNI) interacted with the conserved amino acid Q414 and R419 (Netzler et al., 2017). Despite the bulkiness of CHEMBL1204385 and the presence of carboxylic and amine groups in this compound, only two of the interactions observed in the docking output were preserved (Glu506 and Arg419). The presence of dimethylethanamine and the benzazepine ring in CHEMBL1204385 may have affected the prolific binding of the compound to the protein by altering their backbone conformation. Furthermore, Lys27, an additional residue, formed an H-bond interaction with the carboxylic group of CHEMBL1204385 (Supplementary Figures S5A, B). This implies that the insertion of the sulfonamide group in CHEMBL167790 and PPNDS improves their binding score and interactions. Besides, the presence of the sulfonamide group in CHEMBL167790 will enhance its anti-norovirus inhibitory activity.

Thiophene carboxylic acid derivatives bind at the thumb domain’s outer surface; benzo-1,2,4-thiadiazine derivatives bind to the palm site. Benzimidazoles and indoles which bind to the palm site are the three potent nonnucleoside inhibitors (Le Pogam et al., 2006). CHEMBL167790, the most potent compound, has the structural moiety of thiophene carboxylic acids and is a distinctive polymerase inhibitor class that binds to the allosteric thumb site II of the NS5B protein. The binding pocket of thiophene carboxylic acids is predominantly hydrophobic, and the protein–ligand complexes are stabilized by hydrogen bonding and van der Waals interactions (Biswal et al., 2005). Amide linkages are critical structural elements that maintain an optimal dihedral angle between the amide and thiophene groups. Chan et al. reported the synthesis of 3-arylsulfonylamino-5-phenyl-thiophene-2-carboxylic acid and its anti-HCV activity (Chan et al., 2004). Some of the compounds inhibited HCV NS5B polymerase and HCV subgenomic RNA replication. CHEMBL167790 inhibited HCV NS5B polymerase with an IC_50_ value of 1.0 μM and in the replicon cell-based assay with an EC_50_ value of 5.0 μM. Stephens and co-workers reported synthesizing of 2-amino- and 2-carboxamido-3-arylsulfonylthiophene derivatives and their evaluation against antiviral and antitumor inhibitory activity (Stephens et al., 2001). Among the investigated compounds, 2-amino-3-(2-nitro-phenylsulfonyl)thiophene emerged as the most potent anti-HIV-1 with an EC_50_ value of 3.8 mg/ml, and the observed CC_50_ was >100 mg/ml. In broad-spectrum antiviral assays, some of the tested compounds proved considerably active (IC_50_ = 0.1–10 mg/ml) against human cytomegalovirus (CMV) and/or varicella zoster virus (VZV). Some commercially available drugs containing thiophene moiety include tipepidine, tiquizium bromides, timepidium bromide, dorzolamide, tioconazole, citizolam, sertaconazole nitrate, and benocyclidine. Thus, in searching for a new generation of an anti-norovirus agent with potential pharmacological activities, optimization, and derivation of CHEMBL167790, a derivative of thiophene-2-carboxylic acid can be explored.

### Principal Component Analysis

**FIGURE 7:**
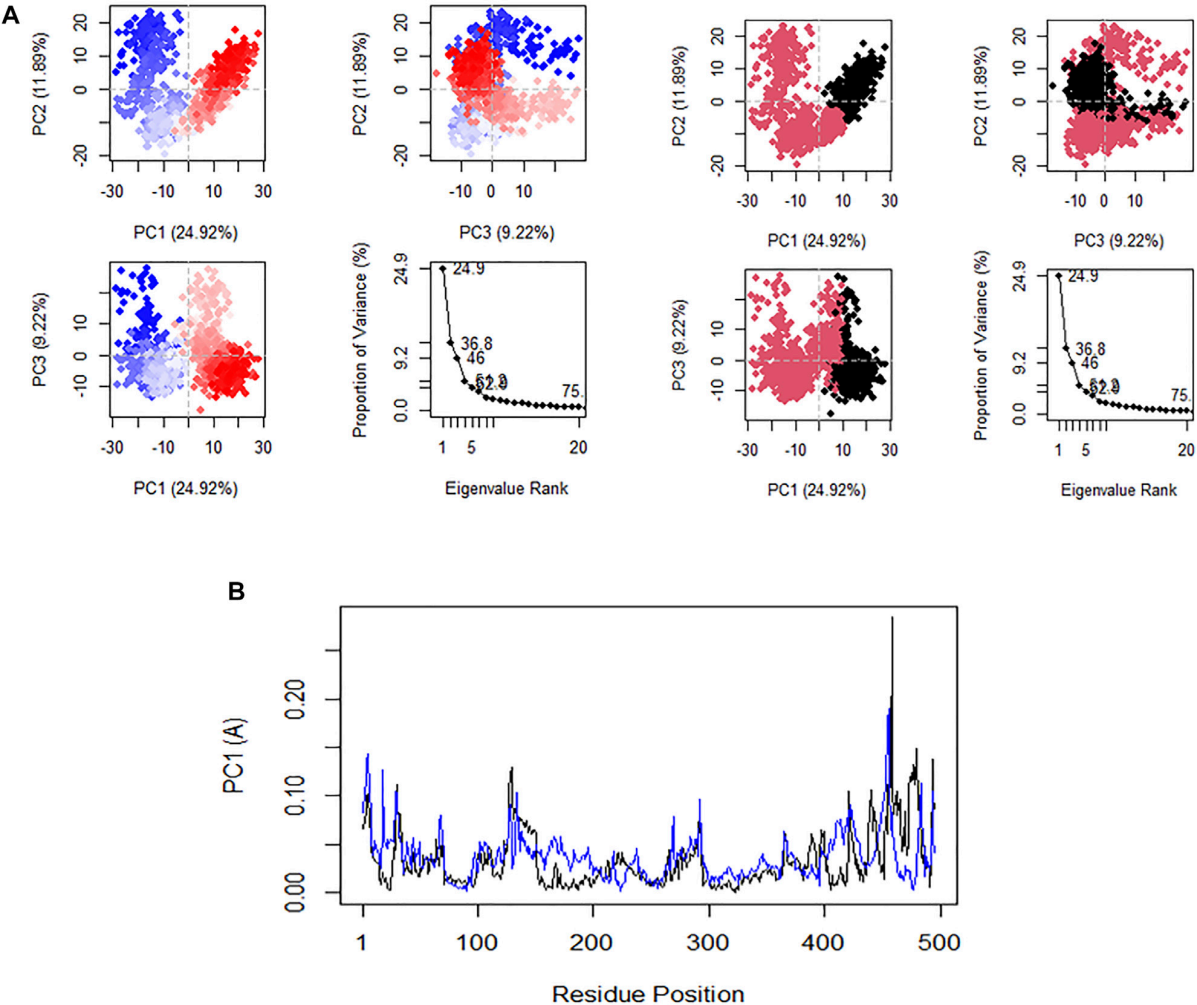
**(A)** Plot of PCA results in an eigenvalue rank: PC2 vs. PC1, PC2 vs. PC3, PC3 vs. PC1, showing color in order of time and the cumulative variability in each data point. **(B)** Residues fluctuated on the PC1 (black) and PC2 (blue).

Principal component analysis (PCA) is very significant because it gives exclusive perception into the nature of clusters and conformational changes in the structure of CHEMBL167790 that result from the MD. The plot of PC1 versus PC2 and PC2 versus PC3 are shown in Figure 7A. The RMSF fluctuation of the residues along the PC1 (black) and PC2 (blue) are shown in Figure 7B. In comparison, the positive or negative value of the eigenvectors of PCA is arbitrary. The regions with the same sign are related in their conformational evolution. In contrast, the regions with opposite signs are related (Mathew et al., 2016). The 20 principal components captured 75.0% of the variance of conformation fluctuations observed in the steady state of CHEMBL167790 during the MD. The first three PCs (PC1, PC2, and PC3) are accountable for 46.01% of the total variance, as seen in the eigenvalue rank plot. PC1 showed the maximum variability (24.9%), followed by PC2 (11.89%), whereas PC3 (9.22%) showed the remaining variability of all the atomic motions through the highest principal components. Notably, the observed constant color change of the PC is specifically from black to deep pink and red to white which indicated a periodic jump during the simulation. We computed the residual displacements along PC1 and PC2 during MD’s equilibrium state, and the RMSF graph was plotted. The plotted graph revealed that the highest fluctuation peak occurred between residues 476 and 475, and these residues are positioned at the loop regions of the protein. There are slight peaks at residues 434 and 436 along PC1 and PC2, respectively. These results are very similar to the RMSF mentioned earlier, thus implying that PC1 and PC2 dominate the conformational fluctuations of HuNoV RdRp and may directly contribute to the inhibitory activity of CHEMBL167790 against norovirus disease.

### Dynamical Cross-Correlation Matrix of the CHEMBL167790–HuNoV RdRp Complex

**FIGURE 8:**
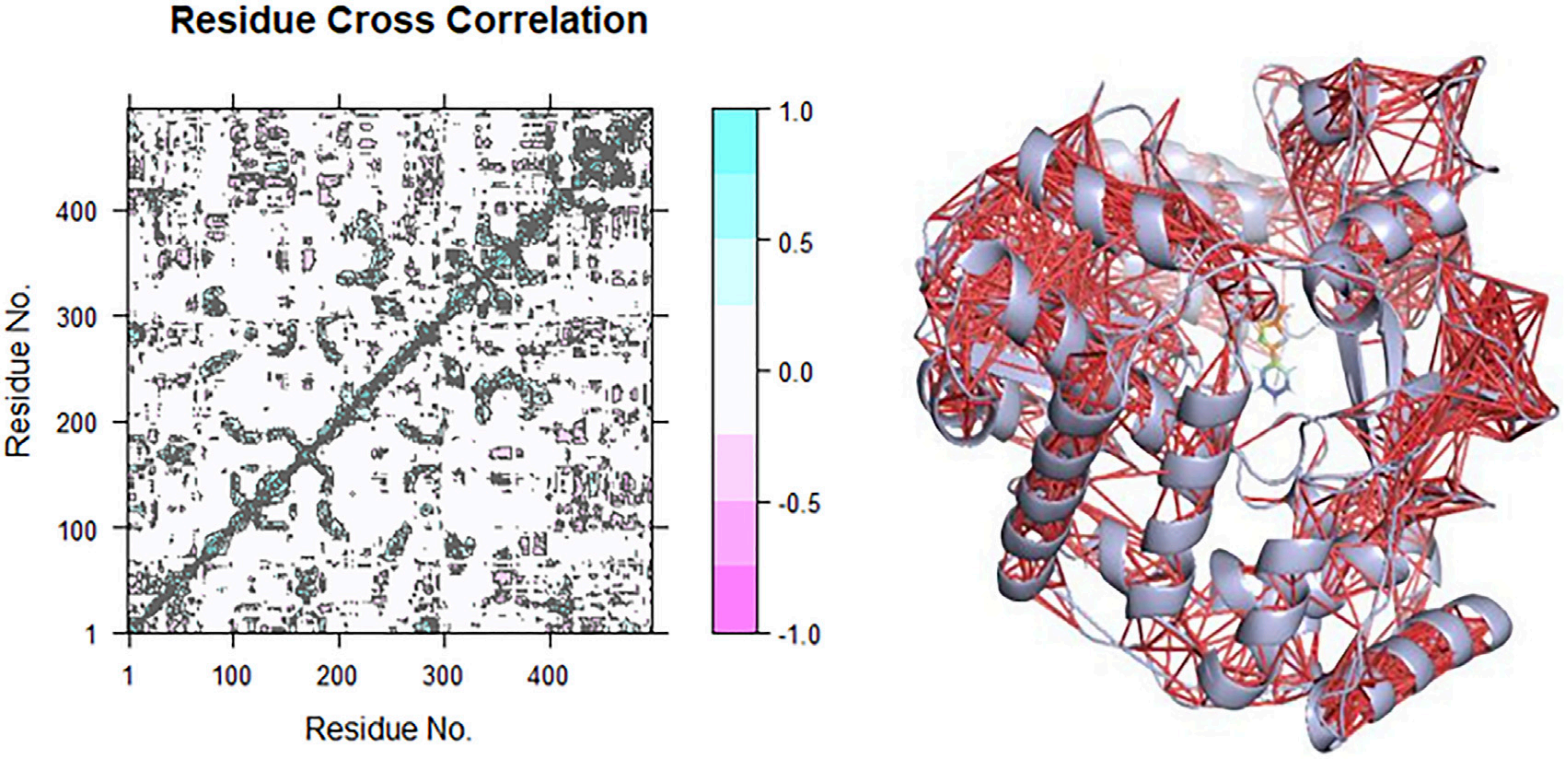
Correlation (red lines) in the residues of the receptors during the MD simulation was obtained using the DCCM method for CHEMBL1204385 interaction with HuNoV RdRp.

The correlation and anti-correlation in the motion of the residues during simulation are shown in Figure 8. The dynamical cross-correlation matrix (DCCM) was used according to

$$C\left(i,j\right)=\;\frac{c\left(i,j\right)}{c{\left(i,i\right)}^{1/2\;}c{\left(j,j\right)}^{1/2}}.$$

C (i,j) is the covariance matrix element of protein fluctuation between residues i and j. The map generated from the result is detailed based on the colors such as dark cyan, white, and pink. The positive area (light and dark cyan) means correlated motions that comprise residues moving in similar directions. In contrast, the negative area (light and deep pink) implies anti-correlation related to residues advancing to reverse directions. The interactions of CHEMBL167790 with HuNoV RdRp depict mostly intra-domain correlation and a small component of inter-domain. There are highly correlated motions nearby the diagonal, as displayed in Figure 8, which stands mainly for the intra-correlations of residues.

### ADMET Detailed of CHEMBL167790

The pharmacological properties of CHEMBL167790 were examined to evaluate its suitability and sustainability for drug development Supplementary Table S1. CHEMBL167790 followed Lipinski’s rule of five for high druggability with no violation. The predicted percentage intestine absorption value of the active ligand (98%) was superior compared to PPNDS (>30%). The intestine is considered a binding site for absorption of a drug from an orally administered solution; hence, high intestine absorption is essential for optimal drug development. But PPNDS is poorly absorbed by the intestine. The predicted caco2 permeability value was >0.9 and skin permeable (log *Kp* of −2.735). Since the brain is inaccessible for exogenous compounds *via* the blood–brain barrier, the ability of the drug to cross the brain is a crucial parameter to consider. The predicted value for BBB (blood–brain barrier) permeability indicated that the active compound could pass through the blood–brain barrier. The predicted BBB permeability (log BB) and CNS permeability (log PS) were 0.508 and −2.243, respectively. The compound showed a low volume of distribution (‒1.453) and was predicted as a non-substrate of the organic cation transporter 2. In the pharmacodynamics studies, the toxicity profile that explained the drug-like compound’s side effects was explored. Toxicity profiling showed that the compound is non-carcinogenic, as predicted by the Ames test. The advancement of the long QT syndrome leading to fatal ventricular arrhythmia is caused by inhibition of the potassium channels encoded by hERG (human ether-à-go-go–related gene). This has resulted in the removal of many medications from the pharmaceutical market. The active compound is predicted to be non-inhibitors of hERG I and II. The results from the study show that the compound has a low value of minnow toxicity (LC50 = ‒0.996) and no skin sensitivity to humans. The maximum tolerated dose predicted was 0.831 (log mg/kg/day), the oral rat acute toxicity (LD50) was 2.806 (mol/kg), and oral rat chronic toxicity (LOAEL) was 1.09 (log mg/kg BW/day). Conceivably, PPNDS displays non-specific inhibitory effects, which was reported by Simeonov et al. (Simeonov et al., 2009). However, due to lack of cell permeability, it was an option out from had been developed into an orally available drug. Meanwhile, CHEMBL167790 has drug-like properties that will improve the superior inhibitory activity of the compound compared to PPNDS and therefore can be developed as drugs available by the oral route. The bioinformatics analysis suggests the possibility of CHEMBL167790 as an anti-norovirus agent. The study has some limitations as the present study has been conducted through extensive bioinformatics analysis. Besides, no comparative studies were conducted to evaluate the effectiveness of the proposed hit compound with the reported anti-norovirus agents. Suitable experimental validations are needed to confirm the therapeutic effectiveness of the hit compound, including the animal model experimentation. Consequently, this study requires further *in vitro* and *in vivo* studies to develop and validate this potential inhibitor of HuNoV RdRp for norovirus infections therapy.

## Conclusion

The drug that can combat human norovirus is still elusive. Different classification models were generated in this study to identify the potential anti-norovirus inhibitors from non-inhibitors utilizing Naïve Bayesian and random forest approaches. The molecular and fingerprint descriptors selected played an essential role in the building of the prediction models. At the same time, the molecular descriptors used in the models could substantially enhance their prediction accuracy. The RF classifier was identified as the best model with an accuracy of 100% for both descriptors. These results indicate that RF classifier enhances the efficiency of virtual screening for HuNoV inhibitors and can be used effectively to identify new HuNoV inhibitor frameworks. The molecular binding of the ligands to the receptor was determined by molecular docking and molecular dynamics simulation analysis. By comparing the Chemgauss4 scores of CHEMBL1204385, CHEMBL167790, and PPNDS, CHEMBL167790 was strongly correlated with the highest negative energy values in the binding pocket of HuNoV RdRp. In addition, CHEMBL167790 binds tightly to the HuNoV RdRp enzyme with excellent stability through RMSD and RMSF analysis. Thus, the MD results show a rare possible event in HuNoV RdRp receptor conformation changes that can significantly favor inhibitory activities of CHEMBL167790 compared with PPNDS. From the evaluation of the pharmacokinetic properties, the pkCSM results detailed that CHEMBL167790 had shown high Caco2 permeability of log *p* > 0.9 and high human intestine absorption of 98%. The higher binding affinity results of CHEMBL167790 with the anti-norovirus target and the pharmacokinetic properties confirmed the effectiveness of anti-norovirus activity in this compound, which would provide impetus to other researchers performing the wet lab and the clinical evaluations.

## Data Availability

The datasets presented in this study can be found in online repositories. The names of the repository/repositories and accession number(s) can be found in the article/Supplementary Material.
