# Supplementary material for: Predicting New Anti-Norovirus Inhibitor With the Help of Machine Learning Algorithms and Molecular Dynamics Simulation–Based Model
Source: Front Chem. 2021 Nov 17;9:753427. doi: 10.3389/fchem.2021.753427 (PMC8636098; doi:10.3389/fchem.2021.753427)
Supplement: Supplementary file 1 [file Table1.DOCX]

| Descriptors | Models | CI | IN | TP | FN | FP | TN | KS | RMSE | MAE | ROC | SP (%) | SE (%) | Q (%) |
| --- | --- | --- | --- | --- | --- | --- | --- | --- | --- | --- | --- | --- | --- | --- |
| External test data | NB | 37 | 3 | 24 | 0 | 3 | 13 | 0.839 | 0.274 | 0.075 | 0.936 | 81.25 | 100 | 92.5 |
|  | RF | 40 | 0 | 27 | 0 | 0 | 13 | 1.000 | 0.100 | 0.060 | 1.000 | 100 | 100 | 100 |
| HCV dataset | NB | 980 | 305 | 582 | 193 | 112 | 398 | 0.517 | 0.487 | 0.237 | 0.828 | 78.04 | 75.10 | 76.26 |
|  | RF | 1283 | 2 | 774 | 1 | 1 | 509 | 0.997 | 0.110 | 0.072 | 1.000 | 99.80 | 99.87 | 99.84 |

***Table S1. Performance of the Naïve Bayes and Random Forest Classifiers for External test set and HCV dataset***


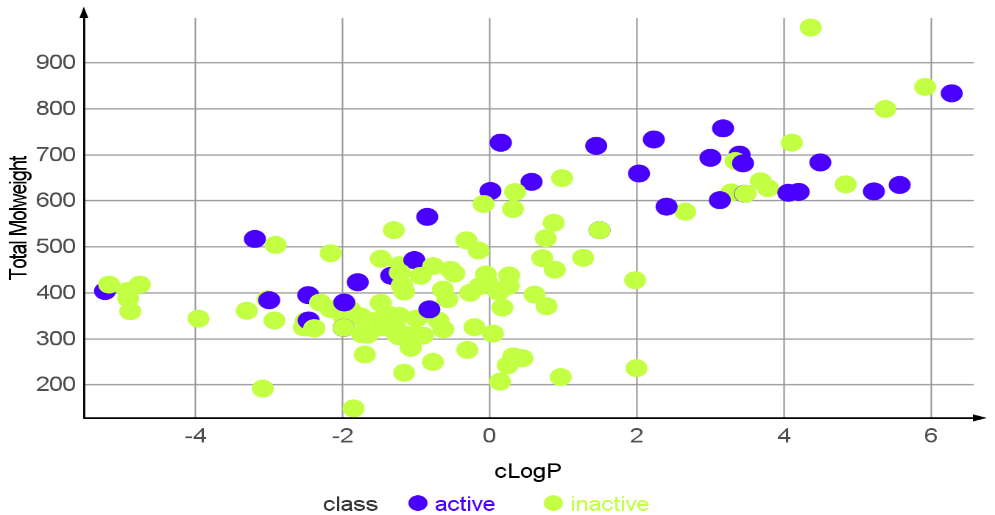


**A**


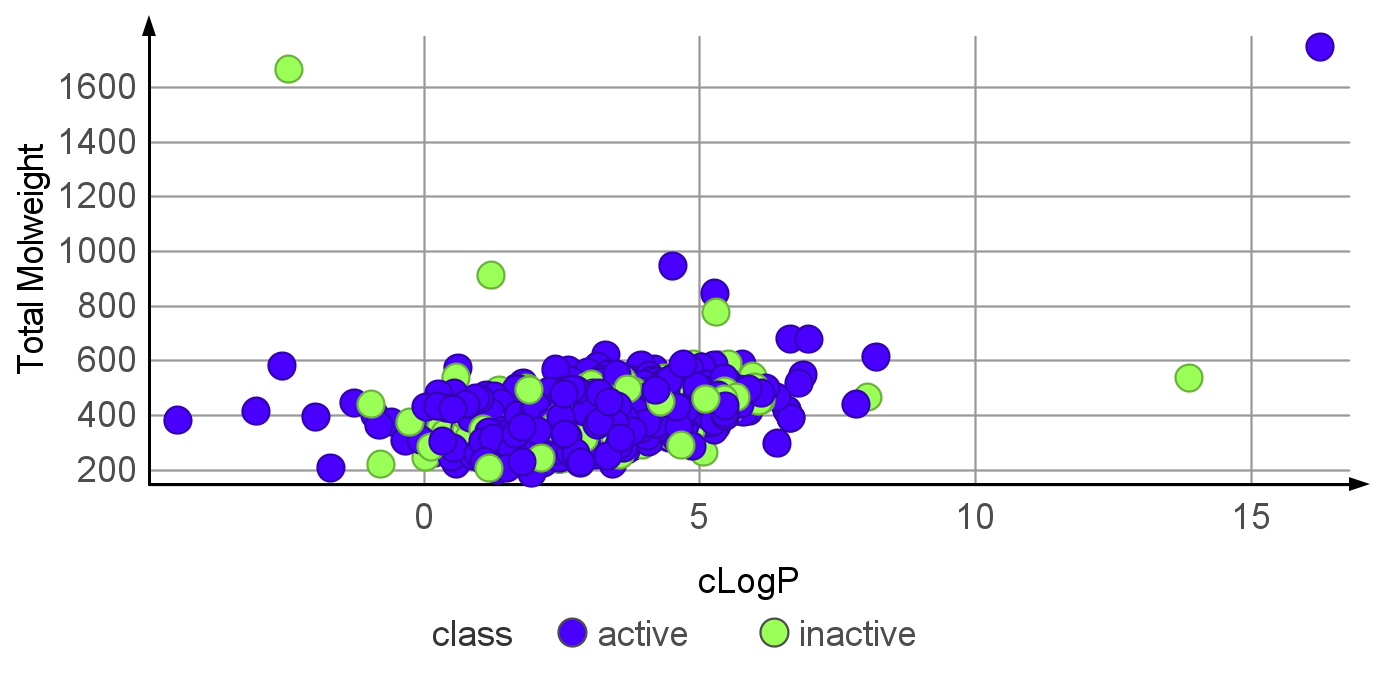


**B**

**Figure S1**. Chemical space distribution of the (A) training set (active and inactive NoV RdRp compounds). (B) Virtual screening set(active and inactive HCV compounds)


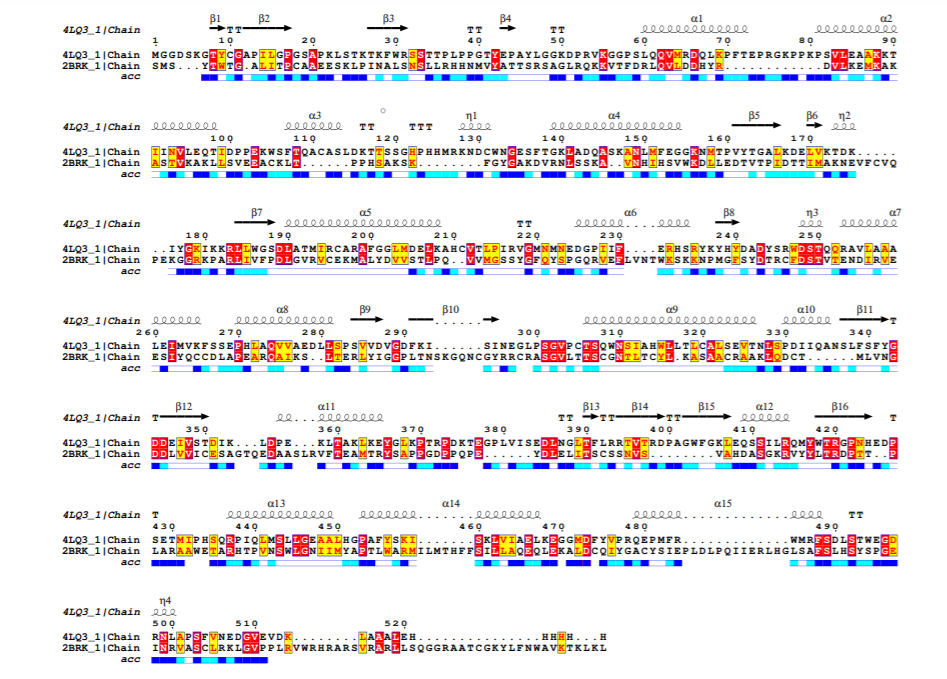


Figure S2. HuNoV and HCV polymerase amino acid alignment; the α-helices and β-strands are shown as coils and arrows, respectively. Disordered regions are shown as dotted lines. The regions that were not included in the polymerase construct are left blank. Strictly conserved residues are colored in red, relative accessibility (blue = accessible, cyan = intermediate, white = buried)


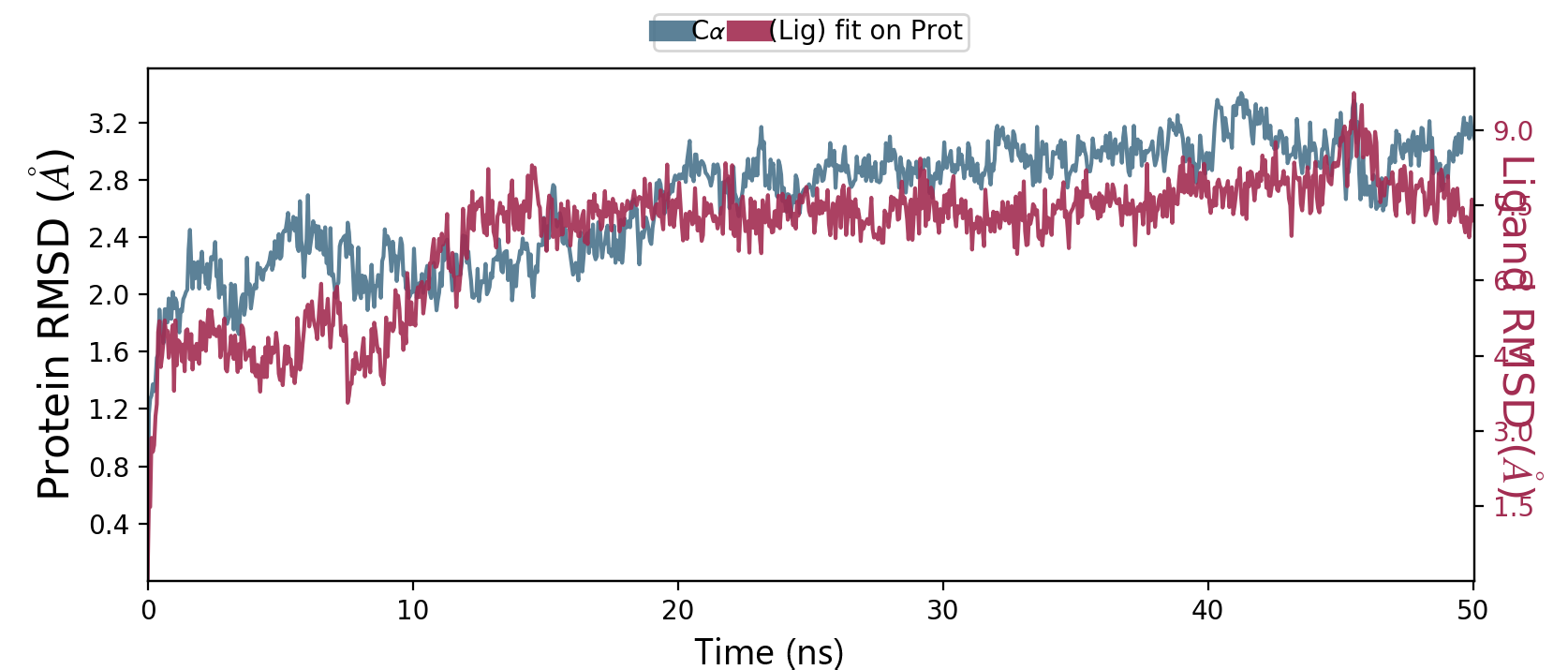


**A**
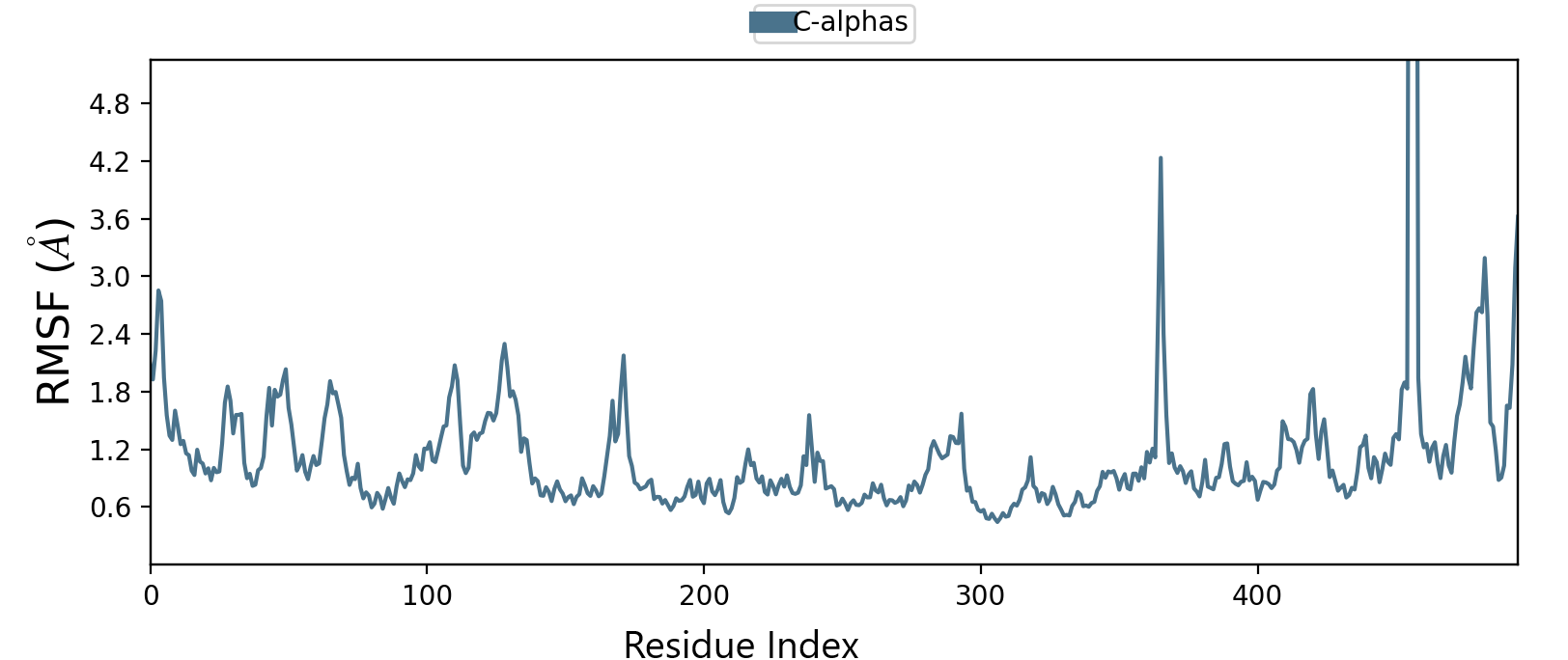


**B**

**Figure S3**. The RMSD and RMSF of the respective complex after molecular dynamics of **CHEMBL1204385**


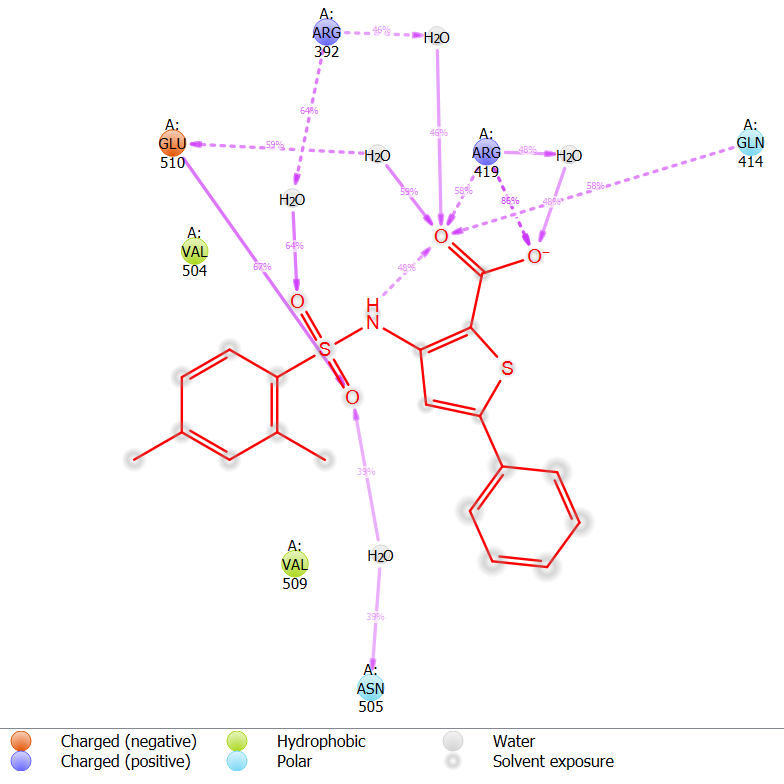


**A**


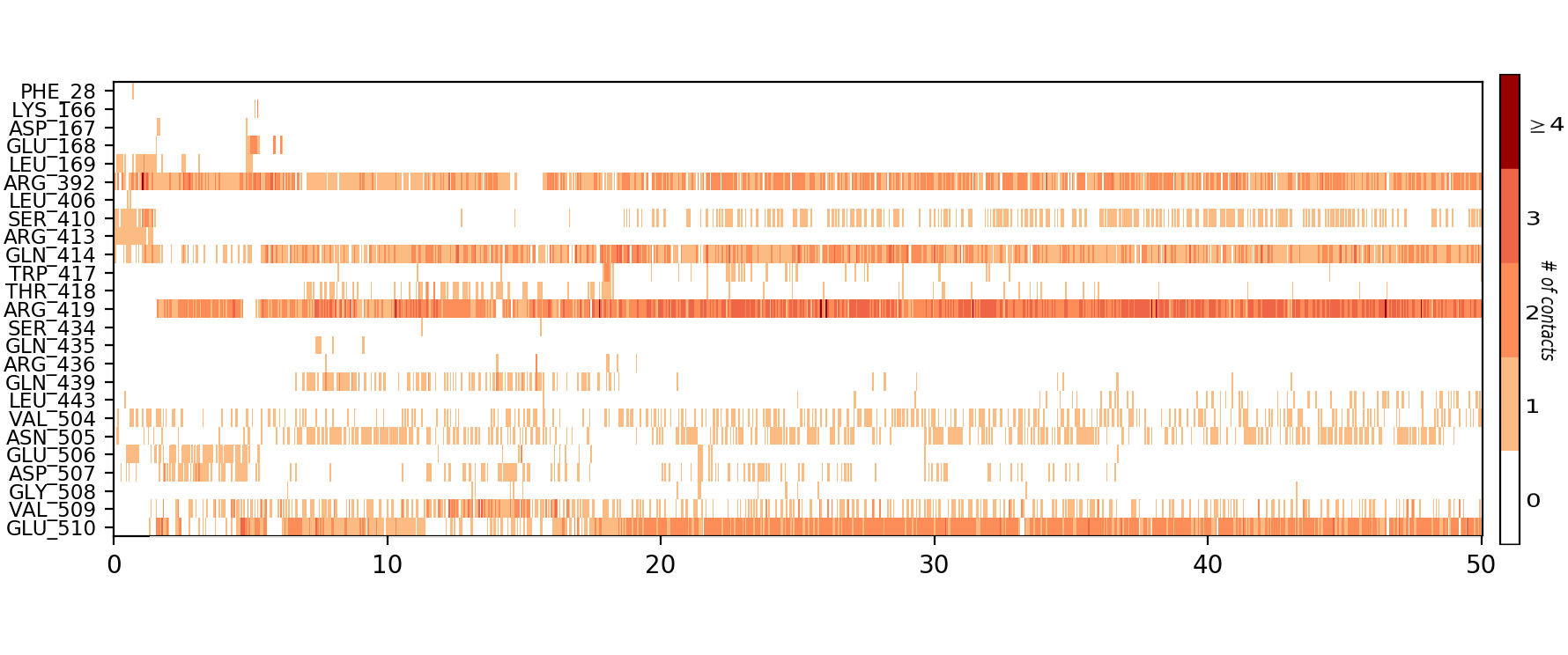


**B**

Figure S4. (A) A schematic of detailed ligand atom interactions with the protein residues of **CHEMBL167790** (B) A timeline representation of the interactions and contacts of **CHEMBL167790**


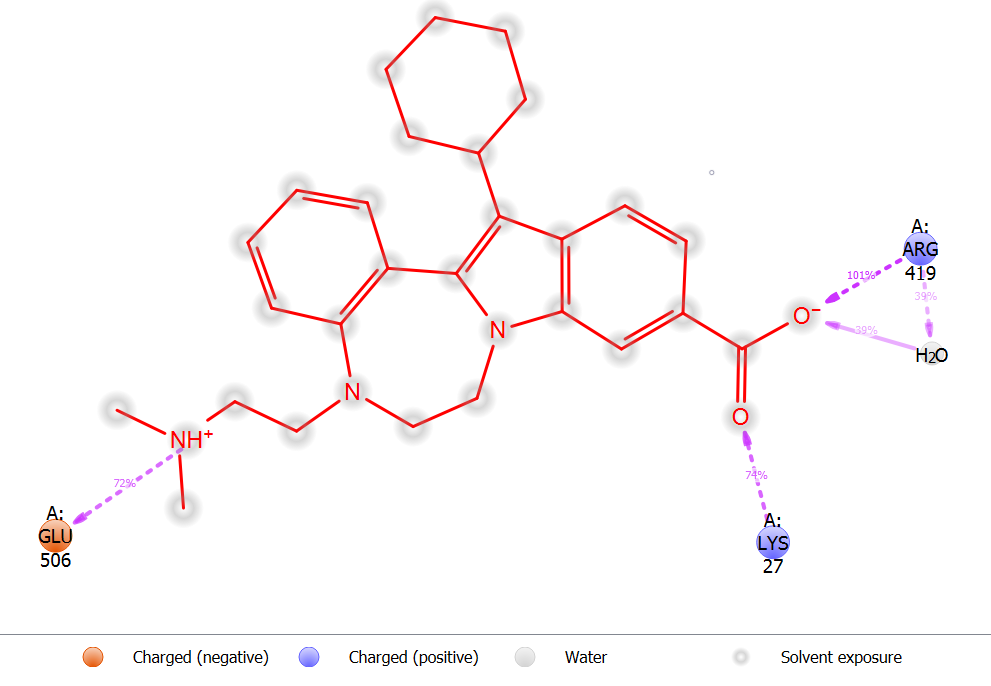


**A**


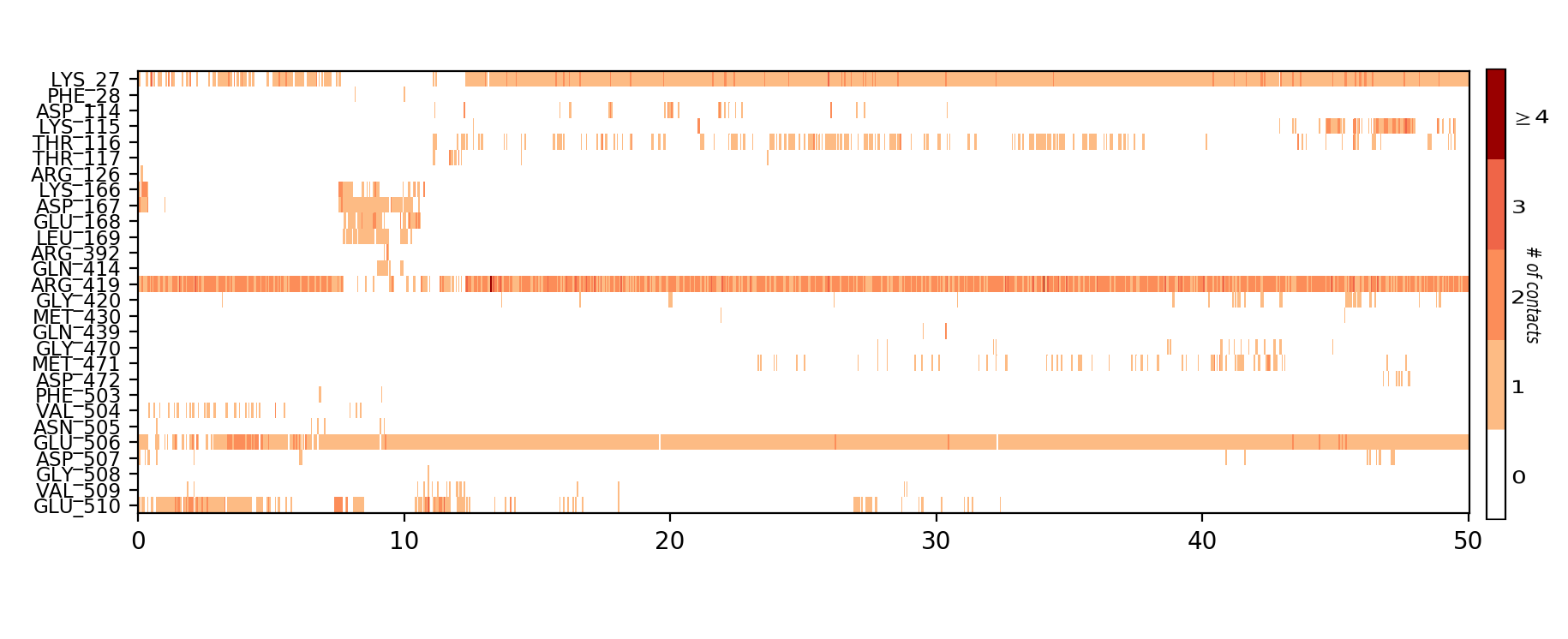


**B**

Figure S5. (A) A schematic of detailed ligand atom interactions with the protein residues of **CHEMBL1204385** (B) A timeline representation of the interactions and contacts of **CHEMBL1204385.**
