# Supplementary material for: Predicting New Anti-Norovirus Inhibitor With the Help of Machine Learning Algorithms and Molecular Dynamics Simulation–Based Model
Source: Front Chem. 2021 Nov 17;9:753427. doi: 10.3389/fchem.2021.753427 (PMC8636098; doi:10.3389/fchem.2021.753427)
Supplement: Supplementary file 2 [file Table2.DOCX]

**[Predicting](https://pubs.acs.org/doi/10.1021/acs.chemrestox.9b00264?ref=vi_computational-toxicology) new anti-norovirus inhibitor with the help of Bayesian machine learning and molecular dynamics simulation-based model**

Oluwakemi Ebenezer*, Nkululeko Damoyi, & Michael Shapi

Faculty of Natural Science, Department of Chemistry, Mangosuthu University of Technology, 511 Mangosuthu Highway, Durban, 4000, South Africa

*****Correspondence: re.korede@gmail.com


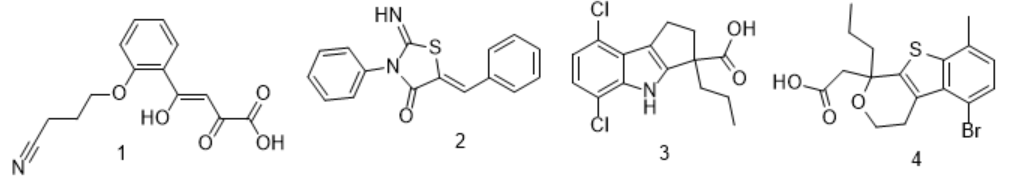

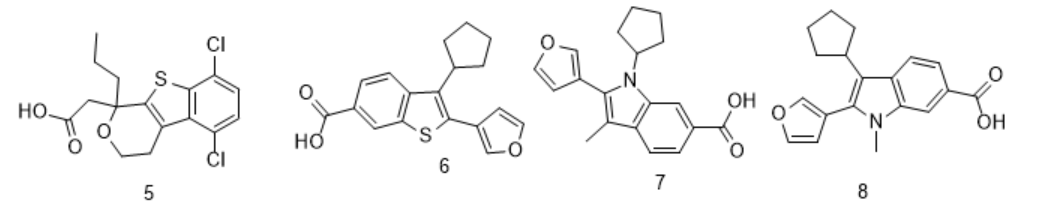


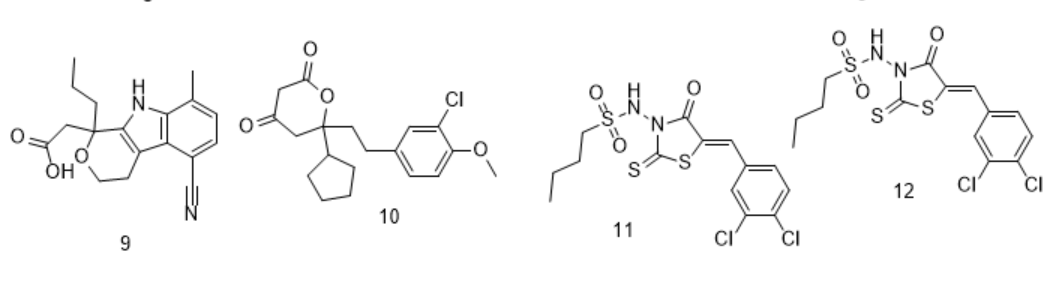

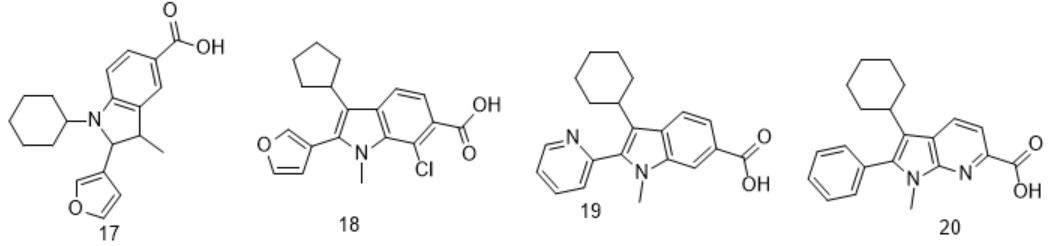


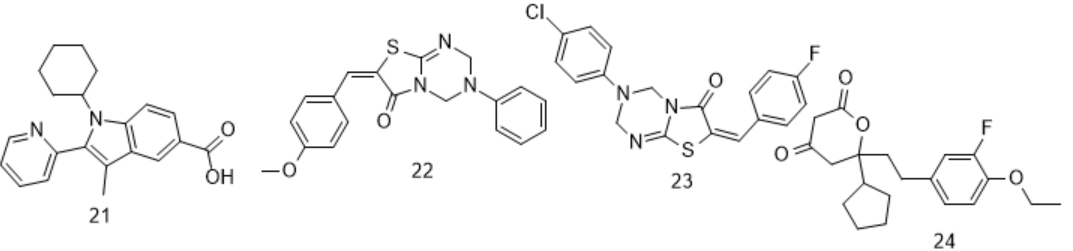

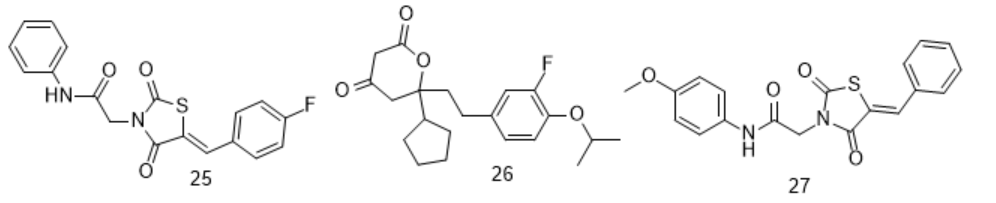


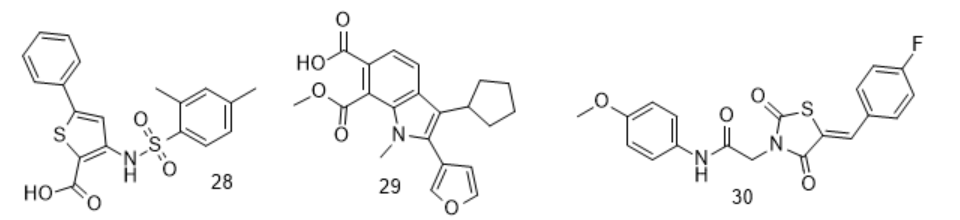

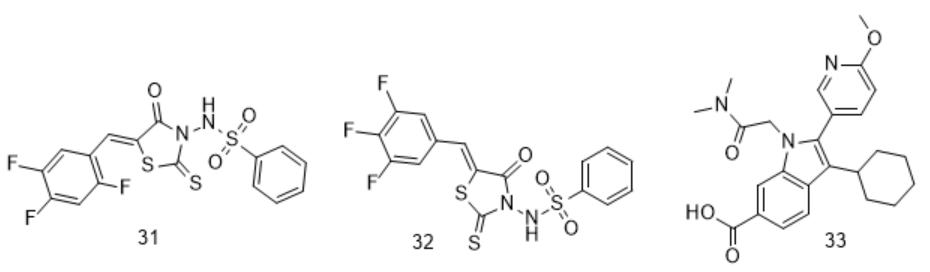


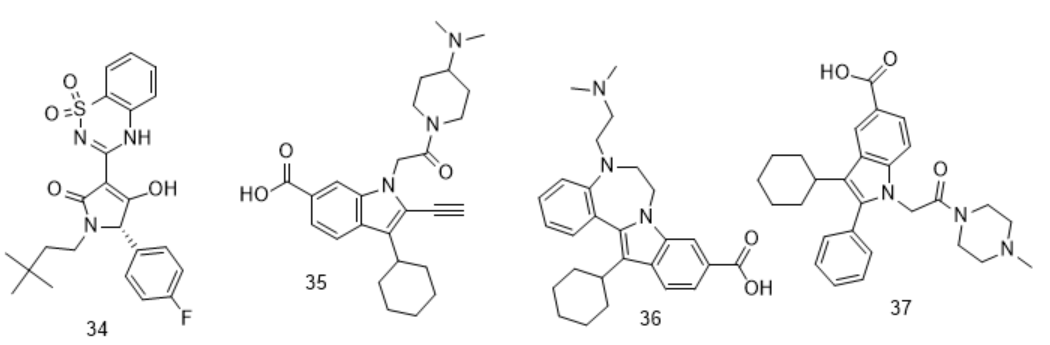

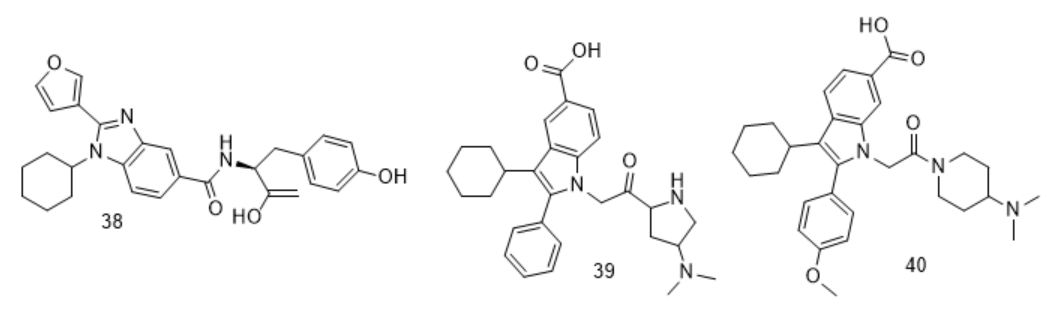


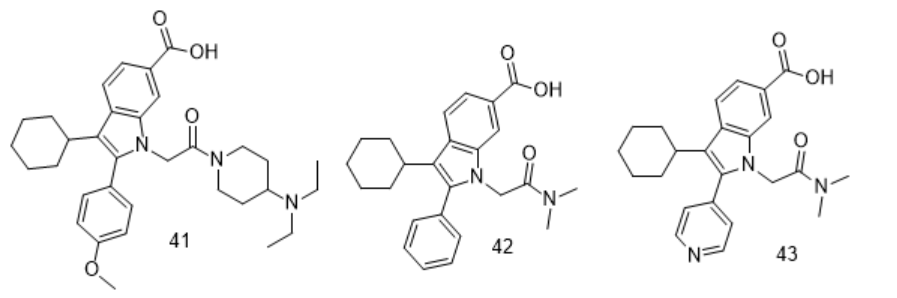

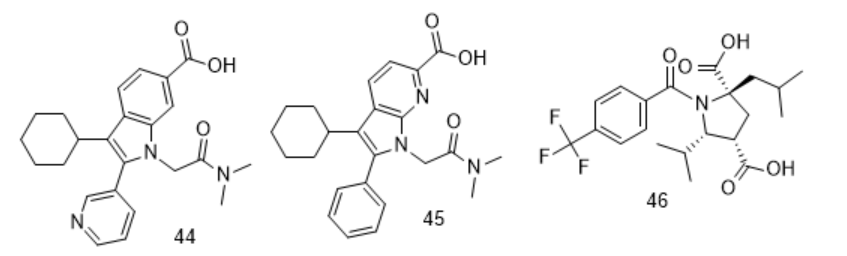


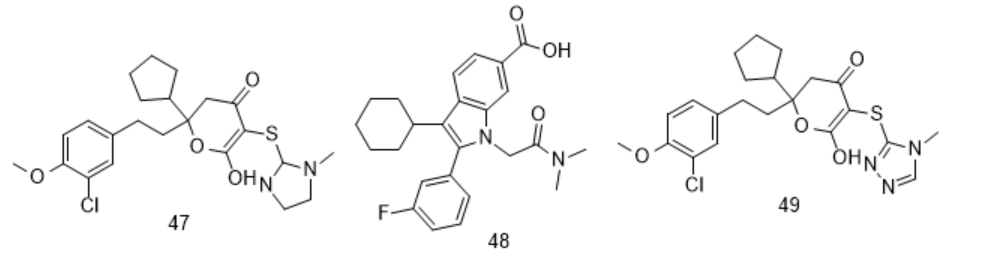

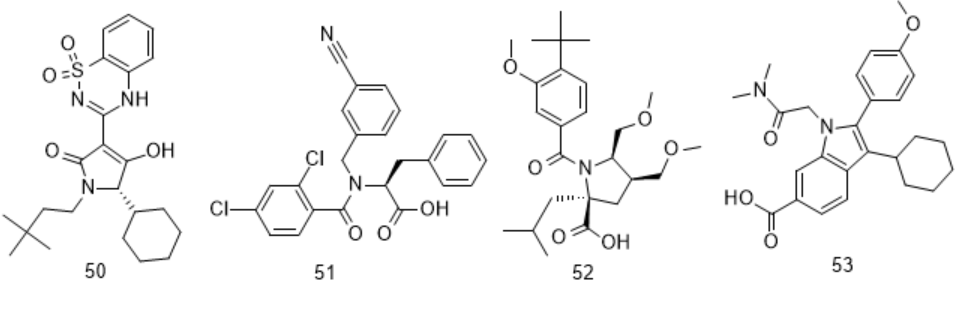


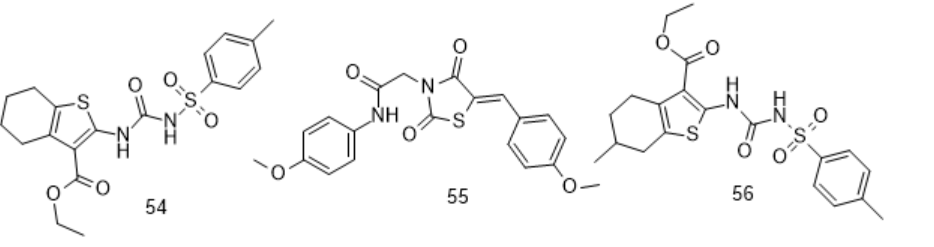

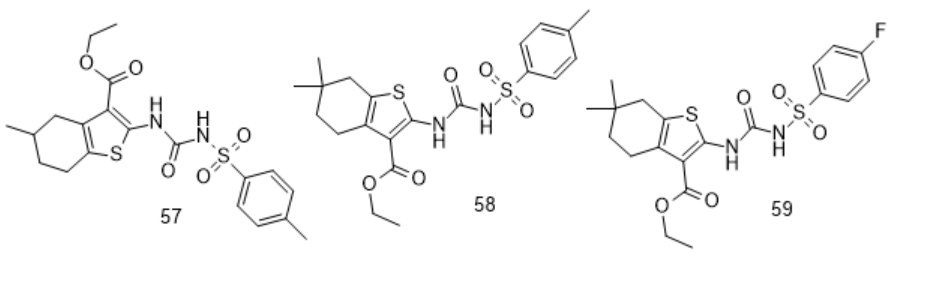


Figure S1. Chemical structures of the screened compounds

Table S1 ADMET properties of the screened compounds

| **Entry** | **Water solubility** | **Caco2** | **Intestinal absorption** | **Skin Permeability** | **BBB permeability** | **CNS permeability** | **Total Clearance** | **Renal OCT2 substrate** | **AMES toxicity** | **hERG I inhibitor** | **hERG II inhibitor** | **Skin Sensitisation** |
| --- | --- | --- | --- | --- | --- | --- | --- | --- | --- | --- | --- | --- |
| Compound 1 | -2.705 | 0.914 | 46.098 | -2.735 | -0.543 | -2.893 | 0.716 | No | No | No | No | No |
| Compound 2 | -4.469 | 1.385 | 92.539 | -2.456 | 0.169 | -1.479 | -0.026 | No | Yes | No | No | No |
| Compound 3 | -3.901 | 1.366 | 96.717 | -2.729 | -0.016 | -1.857 | 0.242 | No | No | No | No | No |
| Compound 4 | -4.63 | 1.416 | 94.675 | -2.727 | 0.376 | -1.672 | 0.189 | No | No | No | No | No |
| Compound 5 | -4.799 | 1.438 | 93.284 | -2.727 | 0.365 | -1.672 | 0.378 | No | No | No | No | No |
| Compound 6 | -4.737 | 1.435 | 91.707 | -2.635 | -0.107 | -1.557 | 0.213 | No | No | No | No | No |
| Compound 7 | -4.252 | 1.376 | 98.357 | -2.727 | 0.393 | -2.688 | 0.945 | No | No | No | No | No |
| Compound 8 | -4.264 | 1.327 | 95.793 | -2.729 | 0.174 | -1.692 | 0.905 | No | No | No | No | No |
| Compound 9 | -3.792 | 0.901 | 95.987 | -2.728 | -0.02 | -2.941 | 0.998 | No | No | No | No | No |
| Compound 10 | -5.539 | 1.349 | 95.045 | -2.74 | 0.298 | -2.108 | 0.058 | No | No | No | No | No |
| Compound 11 | -5.113 | 1.382 | 95.844 | -2.794 | 0.212 | -2.25 | 1.193 | No | No | No | No | No |
| Compound 12 | -5.642 | 0.983 | 89.981 | -3.309 | -0.055 | -2.348 | 0.119 | No | No | No | No | No |
| Compound 13 | -3.625 | 0.991 | 53.953 | -2.735 | -0.492 | -2.534 | 0.648 | No | No | No | No | No |
| Compound 14 | -4.963 | 1.785 | 93.675 | -2.584 | 0.203 | -1.268 | 0.883 | No | No | No | No | No |
| Compound 15 | -4.169 | 1.129 | 94.382 | -2.734 | 0.038 | -1.416 | 0.317 | No | No | No | No | No |
| Compound 16 | -4.341 | 1.351 | 95.964 | -2.73 | 0.147 | -1.6 | 0.905 | No | No | No | No | No |
| Compound 17 | -4.149 | 1.389 | 97.216 | -2.727 | 0.387 | -2.669 | 0.686 | No | No | No | No | No |
| Compound 18 | -4.341 | 1.382 | 94.777 | -2.728 | 0.164 | -1.57 | 0.539 | No | No | No | No | No |
| Compound 19 | -4.442 | 1.374 | 96.836 | -2.729 | 0.227 | -1.614 | 0.787 | No | No | No | No | No |
| Compound 20 | -4.109 | 1.377 | 91.63 | -2.732 | 0.607 | -1.668 | 0.767 | No | No | No | No | No |
| Compound 21 | -4.137 | 1.386 | 98.376 | -2.73 | 0.426 | -2.683 | 0.763 | No | No | No | No | No |
| Compound 22 | -3.85 | 1.092 | 100 | -2.53 | 0.278 | -1.31 | -0.279 | Yes | No | No | No | No |
| Compound 23 | -4.566 | 1.25 | 98.087 | -2.773 | 0.22 | -1.226 | -0.587 | No | No | No | No | No |
| Compound 24 | -5.38 | 1.339 | 95.425 | -2.796 | 0.196 | -2.258 | 1.235 | No | No | No | No | No |
| Compound 25 | -4.485 | 1.074 | 89.591 | -3.035 | -0.141 | -2.753 | -0.221 | No | No | No | No | No |
| Compound 26 | -6.025 | 1.261 | 95.415 | -2.754 | -0.154 | -2.169 | 1.143 | No | No | No | No | No |
| Compound 27 | -4.5 | 1.093 | 90.346 | -3.074 | -0.694 | -2.774 | -0.042 | No | Yes | No | No | No |
| **Compound 28** | **-3.302** | **1.002** | **98.709** | **-2.735** | **-0.121** | **-2.079** | **0.024** | **No** | **No** | **No** | **No** | **No** |
| Compound 29 | -4.467 | 1.062 | 97.687 | -2.735 | -0.249 | -2.137 | 0.917 | No | No | No | No | No |
| Compound 30 | -4.657 | 1.09 | 89.587 | -3.062 | -0.895 | -2.81 | -0.191 | No | Yes | No | No | No |
| Compound 31 | -4.885 | 1.191 | 91.589 | -2.784 | 0.294 | -3.057 | -0.166 | No | Yes | No | No | No |
| Compound 32 | -4.845 | 1.247 | 92.259 | -2.78 | 0.38 | -3.051 | -0.299 | No | Yes | No | No | No |
| Compound 33 | -4.96 | 1.12 | 97.062 | -2.731 | -0.672 | -2.352 | 0.626 | No | No | No | No | No |
| Compound 34 | -4.781 | 1.178 | 95.059 | -2.735 | -1.032 | -2.188 | -0.077 | No | No | No | No | No |
| Compound 35 | -4.731 | 0.974 | 96.629 | -2.732 | -0.309 | -2.275 | 0.605 | No | No | No | No | No |
| Compound 36 | -4.21 | 1.133 | 94.757 | -2.735 | -0.143 | -1.532 | 0.661 | No | No | No | No | No |
| Compound 37 | -3.995 | 1.093 | 95.792 | -2.734 | -0.469 | -2.038 | 0.239 | No | No | No | No | No |
| Compound 38 | -2.892 | 1.035 | 82.993 | -2.735 | -1.056 | -2.295 | 0.683 | No | No | No | No | No |
| Compound 39 | -3.99 | 1.087 | 96.026 | -2.735 | -0.783 | -2.234 | 0.467 | No | No | No | No | No |
| Compound 40 | -4.688 | 1.014 | 96.079 | -2.734 | -0.732 | -2.139 | 0.489 | No | No | No | No | No |
| Compound 41 | -4.008 | 1.084 | 95.37 | -2.733 | -0.916 | -2.132 | 0.407 | No | No | No | No | No |
| Compound 42 | -4.053 | 0.435 | 95.203 | -2.734 | -0.541 | -1.902 | 0.802 | No | No | No | No | No |
| Compound 43 | -4.408 | 1.203 | 96.291 | -2.727 | -0.197 | -2.102 | 0.786 | No | No | No | No | No |
| Compound 44 | -4.483 | 1.164 | 96.07 | -2.726 | -0.197 | -2.097 | 0.784 | No | No | No | No | No |
| Compound 45 | -4.134 | 1.164 | 90.248 | -2.733 | -0.401 | -2.115 | 0.826 | No | No | No | No | No |
| Compound 46 | -2.967 | 0.99 | 43.997 | -2.735 | -1.042 | -2.313 | 0.886 | No | No | No | No | No |
| Compound 47 | -3.535 | 1.246 | 89.664 | -2.735 | -1.071 | -2.18 | 0.75 | Yes | No | No | No | No |
| Compound 48 | -4.087 | 1.021 | 95.439 | -2.732 | -0.7 | -1.915 | 0.688 | No | No | No | No | No |
| Compound 49 | -5.559 | 1.199 | 91.423 | -2.789 | -1.013 | -3.063 | 0.066 | No | No | No | No | No |
| Compound 50 | -4.109 | 1.014 | 89.179 | -2.77 | -0.801 | -1.929 | 0.13 | No | No | No | No | No |
| Compound 51 | -3.614 | 0.907 | 99.11 | -2.735 | -0.128 | -1.869 | 0.176 | No | No | No | No | No |
| Compound 52 | -4.269 | 1.216 | 100 | -2.735 | -0.283 | -3.019 | 0.838 | No | No | No | No | No |
| Compound 53 | -4.947 | 1.062 | 96.577 | -2.731 | -0.393 | -2.087 | 0.477 | No | No | No | No | No |
| Compound 54 | -4.742 | 0.908 | 85.704 | -2.737 | -0.302 | -2.228 | -0.234 | No | Yes | No | No | No |
| Compound 55 | -5.087 | 1.027 | 91.222 | -2.976 | -0.48 | -2.458 | -0.005 | No | No | No | No | No |
| Compound 56 | -4.804 | 1.002 | 85.843 | -2.737 | -0.316 | -2.172 | -0.24 | No | Yes | No | No | No |
| Compound 57 | -4.861 | 0.986 | 85.792 | -2.737 | -0.313 | -2.176 | -0.237 | No | Yes | No | No | No |
| Compound 58 | -4.863 | 1.063 | 86.675 | -2.737 | -0.294 | -2.081 | -0.234 | No | No | No | No | No |
| Compound 59 | -4.405 | 1.14 | 90.462 | -2.735 | -0.502 | -2.825 | -0.323 | No | No | No | No | No |
|  |  |  |  |  |  |  |  |  |  |  |  |  |
|  |  |  |  |  |  |  |  |  |  |  |  |  |
